# Complete chromosome 21 centromere sequencing of families with Down syndrome

**DOI:** 10.1016/j.ajhg.2026.05.010

**Published:** 2026-06-17

**Authors:** F. Kumara Mastrorosa, Alessia Daponte, Luciana de Gennaro, Shu-Cheng Chuang, Jiadong Lin, Yang Sui, David Porubsky, Keisuke K. Oshima, Julie Wertz, Allison N. Rozanski, William T. Harvey, Yanni Wang, Tiffany Y. Leung, Daniel D. Chan, Weiya He, Jordan Knuth, Gage H. Garcia, Marcelo Ayllon, Katherine M. Munson, Kendra Hoekzema, Peter M. Lansdorp, Claudia Rita Catacchio, Mario Ventura, Stephanie L. Sherman, Emily G. Allen, Tracie C. Rosser, Glennis A. Logsdon, Evan E. Eichler

**Affiliations:** 1 Department of Genome Sciences, University of Washington School of Medicine, Seattle, WA 98195, USA; 2 Department of Biosciences, Biotechnology and Environment, University of Bari Aldo Moro, 70124 Bari, Italy; 3 Department of Genetics, Epigenetics Institute, Perelman School of Medicine, University of Pennsylvania, Philadelphia, PA 19104, USA; 4 European Molecular Biology Laboratory (EMBL), Genome Biology Unit, 69117 Heidelberg, Germany; 5 Terry Fox Laboratory, BC Cancer Research Institute, Vancouver, BC V5Z 1L3, Canada; 6 Department of Human Genetics, Emory University School of Medicine, Atlanta, GA 30322, USA; 7 Department of Medical Genetics, University of British Columbia, Vancouver, BC V6T 1Z3, Canada; 8 Howard Hughes Medical Institute, University of Washington, Seattle, WA 98195, USA

**Keywords:** Down syndrome, trisomy 21, centromere, chromosome 21, intellectual disability, aneuploidy, long-read sequencing, methylation, genome assembly, centromere asymmetry

## Abstract

Down syndrome, the most common form of human intellectual disability, results from nondisjunction and an extra copy of chromosome 21 (chr21), also known as trisomy 21 (T21). Small centromeres have been hypothesized to contribute to its etiology, and studies on mice suggest that larger centromeres are more efficiently transmitted, yet complete sequencing of chr21 centromeres has been particularly challenging due to their repetitive nature and homology to chromosome 13. Using long-read sequencing, we sequenced and assembled the centromeres from eight families with a child with T21 (one parent-child trio, six mother-child duos, and one singleton), all resulting from maternal meiosis I errors. A comparison of all proband chr21 centromeres (*n =* 24) to those of control individuals (*n =* 287) shows that small centromeres are not enriched in families with T21 (*p* value = 0.72), contrary to earlier reports. However, chr21 extreme centromere size asymmetry (>10-fold) was observed for two of them. Mothers from these two families with T21 carry some of the smallest chr21 centromeres (143 and 181 kbp) observed in female individuals to date, exhibiting a ∼10.7- and ∼19.4-fold centromeric α-satellite higher-order repeat array size difference between the maternally inherited homologs, respectively. Phylogenetic reconstruction reveals that human chr21 is particularly prone to such asymmetry, with some of the biggest size differences occurring over the last ∼17,000 years of human evolution.

## Graphical abstract

**Figure:**
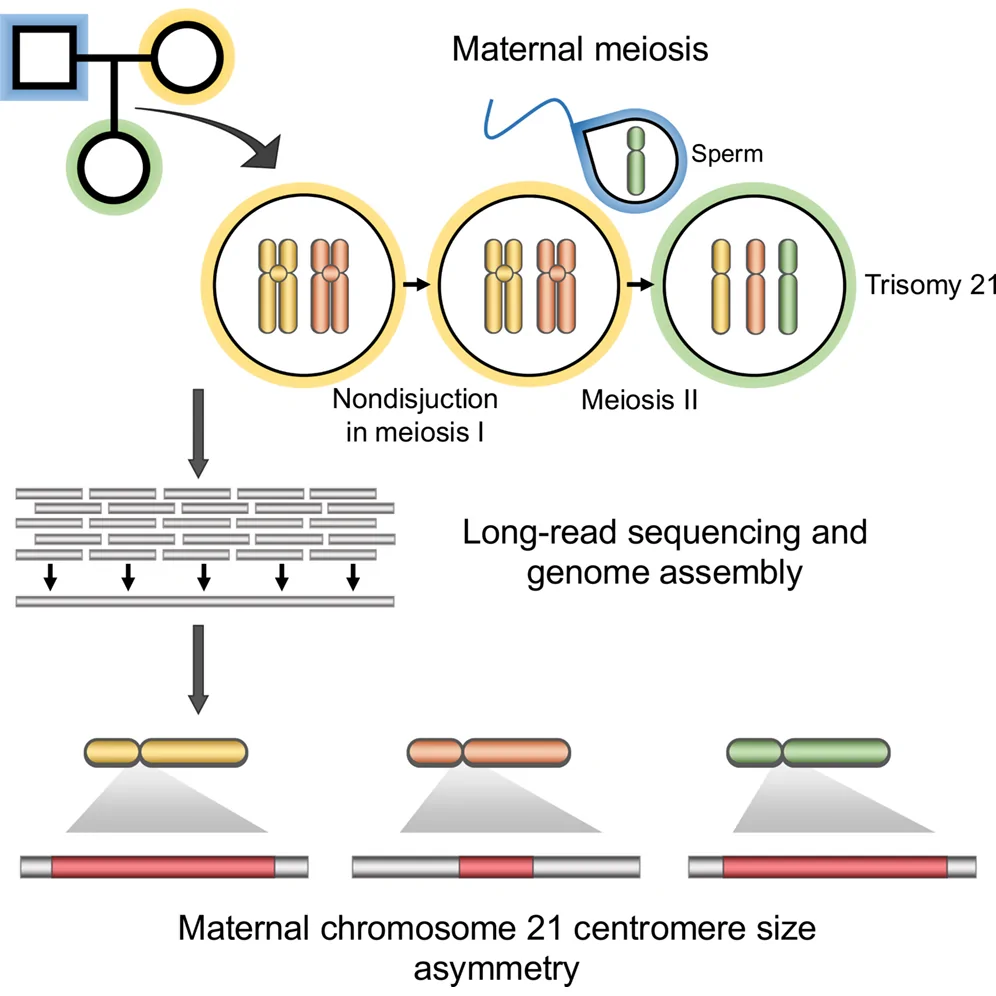

## Teaser

This article describes the genetic and epigenetic landscapes of chromosome 21 centromeres in families with trisomy 21 and the general population. It reveals an extreme size asymmetry of the two maternal chromosome 21 centromeres in a subset of families with affected children and rejects the hypothesis that they generally carry short centromeres.

## Introduction

Trisomy 21 (T21; MIM: 190685) is the most common autosomal chromosomal aneuploidy in humans^1^ and the most common genomic cause of intellectual disability.^2^ The clinical condition, known as Down syndrome, in most affected individuals (>95%) is caused by the presence of an entire extra chromosome 21 (chr21) in the cells of the individual with the syndrome (free T21).^2^ Other cases are caused by a portion or the entire long arm of chr21 translocated to another chromosome as a result of Robertsonian translocations t(14;21) or t(21;21) or mosaic occurrences.^2^ The majority of cases arise as a result of maternal meiosis I errors (MMIEs; ∼66%), followed by maternal meiosis II errors (MMIIEs; ∼21%), paternal meiosis I (∼3%) and meiosis II (∼5%) errors, or mitotic events after the zygote formation (∼5%).^2^

Centromeres play an essential role during meiosis by ensuring equal segregation of homologous chromosomes and sister chromatids. Human centromeric regions consist of different types of satellite DNA, with the most abundant type being α-satellite: a ∼171-bp nearly identical sequence tandemly repeated up to several Mbp in length.^3^ For most human chromosomes, the functional centromere is composed of α-satellite repeats organized into higher-order repeat (HOR) arrays that tend to diverge in sequence at the pericentromeric edges. Until recently, the complete sequence of all human centromeres was unknown because they could not be assembled with standard short-read sequencing data. However, in the last 5 years, improvements in long-read sequencing technologies and computational assembly methods have made it possible to produce several high-quality genome assemblies,^4^^,^^5^^,^^6^^,^^7^^,^^8^^,^^9^^,^^10^^,^^11^^,^^12^^,^^13^ reconstruct the complete sequence of centromeres^12^^,^^13^^,^^14^^,^^15^^,^^16^^,^^17^ and large segmental duplications,^18^^,^^19^^,^^20^ and improve our understanding of the short arm of the acrocentric chromosomes^5^^,^^21^^,^^22^^,^^23^ and other complex loci^5^^,^^24^^,^^25^ previously regarded as inaccessible.

Functionally, each centromere contains a kinetochore, marked by the presence of nucleosomes containing the histone variant H3 CENP-A as well as regions of hypomethylated DNA known as centromere dip regions^15^^,^^16^^,^^26^ (CDRs). Together, these features provide the foundation for the assembly of the kinetochore and play a crucial role in ensuring accurate chromosome segregation during mitosis and meiosis.^27^^,^^28^

Given their role in cell division, centromeres and, in particular, α-satellite HOR array length differences have been postulated to play an important role in chromosome nondisjunction, including Down syndrome.^29^^,^^30^ Studies on mice suggest that centromeres that have more satellite repeats—and stronger kinetochores—are preferentially retained in the egg during maternal meiosis I^31^^,^^32^^,^^33^^,^^34^ and that, in general, centromeric features, such as sequence and length, and kinetochore components^34^^,^^35^^,^^36^^,^^37^ are correlated: a concept known as “centromere strength.” A recent investigation in humans also suggested that individuals with T21 might uniformly have smaller chr21 centromeres (possibly as a result of reduced size of the centromeric α-satellite HOR array).^38^ Centromere size was estimated, however, based on real-time quantitative PCR,^38^ which can be challenging given the high degree of sequence identity between chr13 and chr21 α-satellite DNA and a limited understanding of the sequence diversity within the allelic α-satellite HOR array.^38^ Other studies have shown that cohesion loss between sister centromeres^39^ and reduced recombination^40^ increase the risk of nondisjunction. Nevertheless, the most well-established non-genetic risk factor is increased maternal age at conception,^30^ although there is not a well-established genetic risk factor for free T21.

It is currently unknown whether centromere variation among humans increases the likelihood of nondisjunction. For instance, centromere size, sequence, allelic differences, and epigenetic features might influence nondisjunction risk and indicate predisposition to such events. Recently, combining both Pacific Biosciences (PacBio) high-fidelity (HiFi) long-read sequencing and ultra-long Oxford Nanopore Technologies (UL-ONT) sequencing, we demonstrated that it is possible to completely sequence and distinguish human autosomal alleles for chr21 in diploid individuals,^17^ but this has never been done for a triploid human chromosome as within T21. Here, we present the complete linear sequence and epigenetic profile of all three chr21 centromeres in eight individuals with free T21 caused by MMIE as well as eight of their parents (seven mothers and one father). We also compare genetic and epigenetic features of the chr21 centromeres to a control group of 287 completely sequenced chr21 centromeres from two hydatidiform moles and control individuals (*n =* 156) generated by the Human Genome Structural Variation Consortium^4^^,^^17^ (HGSVC), the Human Pangenome Reference Consortium^6^ (HPRC), and the Arab pangenome effort^11^ to reveal unique features of T21 centromeres.

## Material and methods

### Sample recruitment and data collection

The following cell lines were obtained from the NIGMS Human Genetic Cell Repository at the Coriell Institute for Medical Research: AG16777 (proband), AG16778 (mother), and AG16782 (father). The proband is described as a mosaic with karyotype 47,XX,+21[21]/47,XX,+21,t(21;22)(q22;q13)[29], with 10% of the cells examined showing random chromosome loss and the karyotype confirming the diagnosis. The parents do not have Down syndrome and have a normal karyotype. The mother’s karyotype is 46,XX, with 10% of the cells examined showing random chromosome loss/gain and 2% showing random chromosomal aberrations. The father’s karyotype is 46,XY, with 2% of the cells examined showing random chromosome loss. The proband was 43 years old at sampling, while mother and father were 72 and 74 years, respectively. The proband carries a mosaic translocation between the q-arm of chr21 and chr22 detected in more than half of the cells examined, as reported by Coriell, and at least 12.5% of cells, as determined with Strand sequencing (Strand-seq) (supplemental notes). The translocation does not involve the centromeres and is exclusive of the long arms. Therefore, having focused on the centromeric regions and validated that the proband chr21 centromeres are representative of the maternally transmitted ones, we believe the mosaic translocation does not influence centromere analysis. The maternal karyotype confirms that translocation is exclusive to some cells of the proband, i.e., somatic. This means it must have occurred postzygotically and therefore cannot have influenced the genesis of the trisomy during maternal meiosis, which is instead germline. We obtained lymphoblastoid cell lines from the Emory University biobank for seven probands with MMIE T21 and six mothers. Blood samples were also obtained for the same probands. All study procedures were approved by the Institutional Review Board at Emory University, and written informed consent (and assent when applicable) was obtained from all participants or their legally authorized representatives. Sample 7210079.00, described in this study as carrying chr21 centromere extreme size asymmetry, has a karyotype 47,XY,+21, with no evidence of mosaicism (cells counted: 20; cells analyzed: 6; cells karyotyped: 2; additional scans to confirm phenotype and absent mosaicism: 20).

### PacBio HiFi sequencing

PacBio HiFi data were generated per manufacturer’s recommendations. In brief, high-molecular-weight (HMW) DNA was extracted from frozen blood or cultured lymphoblasts (∼5 × 10^6^ cells) using the Monarch HMW DNA Extraction Kit for Cells & Blood (New England Biolabs [NEB], T3050L). At all steps, quantification was performed with Qubit dsDNA HS (Thermo Fisher, Q32854) measured on DS-11 FX (Denovix) and size distribution checked using FEMTO Pulse (Agilent, M5330AA and FP-1002-0275.) HMW DNA was sheared with Megaruptor 3 (Diagenode, B06010003 and E07010003) using settings 28/31 or 28/30 (depending on original length distribution) and used to generate PacBio HiFi libraries via the SMRTbell Prep Kit 3.0 (PacBio, 102-182-700). Size selection was performed with Pippin HT using a high-pass cutoff of 17 kbp (Sage Science, HTP0001 and HPE7510). Samples AG16778, AG16782, and NG16777 were sequenced on the Sequel II platform on SMRT Cells 8M (PacBio, 101-389-001) using Sequel II Sequencing Chemistry 3.2 (PacBio,102-333-300) with 2-h pre-extension and 30-h movies, aiming for a minimum estimated coverage of 30× in PacBio HiFi reads for parents (3 SMRT Cells per sample) and 60× for the proband (5 SMRT Cells, assuming a genome size of 3.1 Gbp). The remaining samples were sequenced on the Revio platform on SMRT Cells 25M (PacBio, 102-817-900) with diffusion loading and 24-h movies or adaptive loading and 30-h movies, aiming for a minimum estimated coverage of 30× in PacBio HiFi reads for the parents (1–1.5 SMRT Cells per sample) and 60× for the probands (2–2.5 SMRT Cells, assuming a genome size of 3.1 Gbp).

### UL-ONT sequencing

Ultra-HMW genomic DNA (gDNA) was extracted from the lymphoblastoid cell lines according to a previously published protocol.^41^ In brief, 1–3 × 10^7^ cells were lysed in a buffer containing 10 mM Tris-Cl (pH 8.0), 0.1 M EDTA (pH 8.0), 0.5% (w/v) SDS, and 20 mg/mL RNase A for 1 h at 37°C. 200 μg/mL proteinase K was added, and the solution was incubated at 50°C for 2 h. DNA was purified via two rounds of 25:24:1 phenol/chloroform/isoamyl alcohol extraction followed by ethanol precipitation. Precipitated DNA was solubilized in 10 mM Tris (pH 8.0) containing 0.02% Triton X-100 at 4°C for 2 days. Libraries were constructed using the Ultra-Long DNA Sequencing Kit (ONT, SQK-ULK001 or SQK-ULK114) following the manufacturer’s protocol. Next, 75 μL of library was loaded onto primed FLO-PRO002 R9.4.1 or FLO-PRO114M R10.4.1 flow cells for sequencing on the PromethION, with two nuclease washes and reloads after 24 h and 48 h of sequencing. Sequence reads > 100 kbp in length were classified as ultra-long (UL). All ONT data were basecalled using guppy (v.6.3.7 or newer) or dorado (v.0.4.2 or newer) with the SUP model.

### Genome assembly

Proband AG16777 genome assembly was generated with the Verkko^42^ (v.1.2) (https://github.com/marbl/verkko) workflow using HiFi sequencing data at ∼59× and UL-ONT (>100 kbp) data at ∼23× coverage following the developer instructions. Parental assemblies (AG16778 and AG16782) were generated with both Verkko^42^ (v.1.4) (https://github.com/marbl/verkko) and hifiasm^43^ (v.0.19.5) (https://github.com/chhylp123/hifiasm) using HiFi data at ∼37× for the mother and 40× for the father and UL-ONT data at 25× and 12×, respectively. HiFi developer instructions were followed. Similarly, genome assemblies for the second cohort of probands with T21 and their mothers were generated using Verkko^42^ (v.2.0 or v.2.1) and hifiasm^43^ (v.0.19.8 or v.0.19.9). To assemble the highest number of chr21 centromeres possible, we assembled the genomes of the families with T21 using both Verkko^42^ and hifiasm,^43^ testing several versions ranging from 1.2 to 2.1 for Verkko^42^ and from 0.19.5 to 0.19.9 hifiasm.^43^ Although the basis for this is unknown, analysis of HGSVC samples has shown that use of both assemblers yields the highest number of completely assembled centromeres.^12^ Therefore, we retained for analysis the versions containing the most complete and accurate chr21 centromeres and, in a few cases, we combined the results from the two assemblies (see contig fixing and patching).

### Cell culture, slide preparation, and immunofluorescence combined with fluorescence *in situ* hybridization

AG16777, AG16778, and AG16782 cells were cultured in RPMI-1640 medium supplemented with 16% fetal bovine serum, 1% L-glutamine, and 1% penicillin-streptomycin. Metaphase and interphase nuclei undergoing immunofluorescence with mouse CENP-A monoclonal antibody (Enzo, ADI-KAM-CC006-E) were prepared by blocking the cells in metaphase with colcemid, followed by washing in 1× PBS. The cells were then counted and resuspended in a hypotonic buffer (75 mM KCl/0.8% NaCitrate/3 mM CaCl_2_/1.5 mM MgCl_2_, 1:1:1:1 ratio) for 15 min to reach a final concentration of 5 × 10^5^ cells/mL. A 0.5-mL aliquot of the cell suspension was then cytocentrifuged onto Superfrost Plus slides at 1,500 rpm for 5 min using a Shandon Cytospin 3 (Thermo Scientific). The slides were immediately used for immunofluorescence. For slides undergoing immunofluorescence with mouse CENP-C monoclonal antibody (Abcam, ab50974), colcemid-treated cells were incubated in hypotonic solution (0.56% KCl) for 30 min and prefixed by adding methanol/acetic acid (3:1) fixative solution. Prefixed cells were collected by centrifugation and fixed in methanol/acetic acid (3:1). Spreads were dropped on a glass slide and incubated at room temperature overnight.

Immunofluorescence was performed on the metaphase spreads using mouse CENP-C and CENP-A monoclonal antibodies as previously described with minor modifications.^44^ In brief, each slide was rehydrated by immersion in 1× PBS-azide (10 mM NaPO_4_ [pH 7.4], 0.15 M NaCl, 1 mM EGTA, and 0.01% NaN_3_) for 15 min at room temperature. Chromosomes were then swollen by washing the slides (three times, 2 min each) with 1× TEEN (1 mM triethanolamine-HCl [pH 8.5], 0.2 mM NaEDTA, and 25 mM NaCl), 0.5% Triton X-100, and 0.1% BSA. The primary antibodies were diluted to a final concentration of 0.001 μg/μL in the same solution and then added (100 μL) onto the slides. Each slide was incubated for 2 h at 37°C. Excess of primary antibody was removed by washing the slides at room temperature (three times, 2, 5, and 3 min each) with 1× KB buffer (10 mM Tris-HCl [pH 7.7], 0.15 M NaCl, and 0.1% BSA). A goat anti-mouse immunoglobulin G (IgG) secondary antibody conjugated to fluorescein isothiocyanate (Abcam, ab6785) was diluted 1:100 in the same solution, and 100 μL was added to the slides that were then incubated for 45 min at 37°C in a dark chamber. After incubation with the secondary antibody, the slides were washed once with 1× KB for 2 min, prefixed with 4% paraformaldehyde in 1× KB for 45 min at room temperature, washed with dH_2_O by immersion for 10 min at room temperature, and fixed with methanol/acetic acid (3:1) for 15 min. Fluorescence *in situ* hybridization (FISH) was then performed using a plasmid containing the α-satellite sequence shared between chromosomes 13 and 21 (pZ21A) directly labeled by nick translation with Cy3-dUTP (Enzo, 42501) according to a standard procedure with minor modifications.^45^ In brief, 100 ng of labeled probe was used for the FISH experiments; DNA denaturation was performed at 70°C for 8 min and hybridization at 37°C in 2× SSC, 50% (v/v) formamide, 10% (w/v) dextran sulfate, and 3 mg of sonicated salmon sperm DNA, in a volume of 10 μL. Post-hybridization washing was performed under high-stringency conditions: at 60°C in 0.1× SSC (three times, 5 min each). Nuclei and chromosome metaphases were simultaneously DAPI stained. Digital images were obtained using a Leica DMRXA2 epifluorescence microscope equipped with a cooled CCD camera (Princeton Instruments). DAPI, Cy3, and fluorescein fluorescence signals, detected with specific filters, were recorded separately as grayscale images. Pseudocoloring and merging of images were performed using ImageJ (v.1.53k). The CENP-C fluorescence intensity at chr21 centromeres was measured on 13 metaphases for each sample using a modified version of the ImageJ macro, CRaQ.^46^ Signals from chr21 were isolated, and a 7 × 7 pixel box was placed around the centroid position of each signal. The pixel intensity within each box was measured and normalized, dividing it by the lower chr21 signal intensity for each analyzed metaphase. Plots were then obtained using the ggplot2^47^ package in R.^48^

### CENP-A DiMeLo-seq

CENP-A DiMeLo-seq using ONT long-read sequencing was performed by modifying the original DiMeLo-seq protocol^49^ and the FindingNemo gDNA extraction and ONT library preparation protocols.^50^ The final protocol was described in Gao et al.^51^ In brief, 7 × 10^6^ cells were resuspended in 1 mL of Dig-Wash buffer (20 mM HEPES-KOH [pH 7.5], 150 mM NaCl, 0.5 mM spermidine, 1 tablet of cOmplete EDTA-free protease inhibitor cocktail [11873580001, Roche] per 50 mL of buffer, 0.1% [w/v] BSA, and 0.02% Digitonin) and incubated on ice for 5 min to isolate nuclei. Nuclei were pelleted and resuspended in 200 μL of Tween-Wash buffer (20 mM HEPES-KOH [pH 7.5], 150 mM NaCl, 0.5 mM spermidine, 1 tablet of cOmplete EDTA-free protease inhibitor cocktail per 50 mL of buffer, 0.1% [w/v] BSA, and 0.1% [w/v] Tween 20) containing a primary antibody at a 1:50 dilution. Primary antibodies used in this study were mouse anti-CENP-A antibody (ADI-KAM-CC006, Enzo) or rabbit anti-IgG antibody (ab171870, Abcam). Samples were rotated overnight on a nutator at 15 rpm and 4°C to allow for antibody binding. A mouse nanobody-Hia5 (Nb-Hia5) from the Altemose lab^52^ was prepared as a secondary antibody at 200 nM in 200 μL of Tween-Wash buffer. After washing away unbound primary antibody with Tween-Wash buffer, samples were resuspended in secondary antibody buffer and rotated for 2 h on a nutator at 15 rpm and 4°C. The methyl donor *S*-adenosyl methionine (SAM; B9003S, NEB) was prepared at 800 μM in 100 μL of activation buffer (15 mM Tris [pH 8.0], 15 mM NaCl, 60 mM KCl, 1 mM EDTA [pH 8.0], 0.5 mM EGTA [pH 8.0], 0.05 mM spermidine, and 0.1% [w/v] BSA). After washing away unbound Nb-Hia5, samples were resuspended with SAM activation buffer and incubated at 37°C for 2 h to activate the DNA adenine methyltransferase activity of Hia5. Following incubation, samples were collected and resuspended in 400 μL of cold PBS for gDNA extraction using the Monarch HMW DNA Extraction Kit for Cells & Blood (T3050S, NEB) by following the FindingNemo protocol.^50^ Extracted gDNA was kept at room temperature for at least 48 h to improve DNA homogeneity. For ONT library preparation, the FRA (transposase fragmentation mix) in the Ultra-Long DNA Sequencing Kit V14 (SQK-ULK114, ONT) was adjusted by adding 8 μL for optimal DNA fragmentation, and library DNA was eluted in 100 μL of 10 mM Tris-HCl (pH 9.0) for a single library load, following the modified FindingNemo protocol.^51^ The DNA library was loaded onto a PromethION 24 R10.4.1 flow cell (FLOPRO114M, ONT) and sequenced with MinKNOW v.25.03.7 (ONT). To enrich centromeric sequences during sequencing, we used the deplete mode of adaptive sampling enabled in MinKNOW to exclude sequencing reads originating from outside centromeric regions. The depletion BED file was made based on centromere annotations of the T2T-CHM13v2.0 reference genome. Libraries were retrieved and reloaded after 20–24 h, depending on pore availability. To obtain sufficient coverage, two DNA libraries were prepared for some samples.

Both anti-CENP-A and anti-IgG DiMeLo-seq ONT data were basecalled with dorado v.0.9.6 + 0949eb8d using the following parameters and model: sup,5mCG_5hmCG,6mA --min-qscore 10; basecall_model = dna_r10.4.1_e8.2_400bps_sup@v5.0.0,modbase_models = dna_r10.4.1_e8.2_400bps_sup@v5.0.0_5mCG_5hmCG@v3, dna_r10.4.1_e8.2_400bps_sup@v5.0.0_6mA@v3. ONT reads were aligned to their respective assemblies using minimap2^53^ (v.2.28) with following parameters: -y -a --eqx --cs -x lr:hqae - I8g -s 4000. To quantify *N*^6^-methyladenine (6mA) methylation, we applied modkit (v.0.3.2, https://github.com/nanoporetech/modkit) using the pileup command with the parameters modkit pileup {input.bam} {output.bed} --ref {input.fa} --motif AT 0 --filter- threshold 0.99 to filter out potentially spurious methylation calls. Finally, using a custom pipeline, Snakemake-DiMeLo-seq-Viz (commit e86297b14a92539dc755a52357b1e778ef506d0b, https://github.com/logsdon-lab/Snakemake-DiMeLo-seq-Viz), we binned each 6mA window into 5-kbp nonoverlapping windows, calculated the average 6mA signal, and divided the average CENP-A signal by the average IgG signal to determine the fold enrichment of CENP-A over IgG per sample. Any invalid values like “Inf” were removed prior to visualization and downstream analysis.

### Strand-seq data generation and analysis

We generated Strand-seq data from lymphoblastoid cell lines for trio AG167. After culture with bromodeoxyuridine (BrdU) for 24 h, formaldehyde-fixed nuclei with BrdU in the G_1_ cell-cycle phase were enriched by fluorescence-activated cell sorting after staining with Hoechst 33258 and propidium iodide, as previously described.^54^ Approximately 80 single-cell Strand-seq libraries were generated for each sample. Single-nuclei isolation and nanoliter reagent dispensing into the wells of a 5,184-nanowell array was performed as described previously.^13^^,^^54^ In brief, formaldehyde crosslinking of nuclei was reversed by heat, and structural nuclear proteins were digested by a heat-labile protease. Restriction enzymes AluI and HpyCH4V (NEB) were used for fragmenting gDNA, and Hemo KlenTaq (NEB) was used for A-tailing. Fragments were ligated to forked adapters, UV irradiated, and PCR amplified with index primers prespotted into nanowells before library preparation. Strand-seq libraries were size selected with AMPure XP beads and 300- to 700-bp fragments were gel purified prior to PE75 sequencing on the NextSeq 550 (Illumina). Next, demultiplexed FASTQ files were aligned to the T2T-CHM13 human genome reference (hs1) using BWA^55^ (v.0.7.17-r1188). Aligned reads were sorted by genomic position using SAMtools^56^ (v.1.10), and duplicate reads were marked using sambamba^57^ (v.1.0). Strand-seq libraries used in subsequent analysis were manually selected to exclude libraries with an uneven coverage or an excess of “background reads” as previously described.^58^ This is done to ensure accurate structural variant (SV) detection and phasing. Lastly, we processed selected libraries using breakpointR (https://github.com/daewoooo/breakpointR) (v.1.18.0) to detect sister chromatid exchanges within each cell as well as recurrent changes in strand state that were indicative of previously described reciprocal translocation between chromosomes 21 and 22p.^59^ This allowed us to map recombination breakpoints in the proband (supplemental notes) as previously described.^13^

### Centromere characterization and validation

Assemblies of individuals with T21 and from the general population were aligned against the T2T-CHM13 (v.2.0) reference genome with minimap2^53^ (v.2.24) (https://github.com/lh3/minimap2) using the following options “-t {threads} -I 15G -a –eqx -x asm20 -s 5000.” For fasta sequences from the contigs mapping against the chr21 centromere (chr21:10700000–11650000), we considered contigs correctly representing chr21 centromeres as those that mapped to the centromere and extended into the q-arm pericentromere. Those contigs mapping to the centromere and to the p-arm, but breaking before the q-arm, were considered mismapped and therefore excluded. RepeatMasker^60^ (v.4.1.0) was used to identify the regions containing the α-satellite array (marked as “ALR/Alpha”) and HumAS-HMMER (https://github.com/fedorrik/HumAS-HMMER_for_AnVIL) to determine the α-satellite HOR array composition with the following command: hmmer-run.sh {path_to_directory_with_fasta} AS-HORs-hmmer3.0-170921.hmm {threads}. For the contigs aligning to the reference genome in the minus orientation, we converted the fasta sequences to their reverse complement before running the analysis. The outputs from HumAS-HMMER analyses were visualized with R^48^ (v.4.2.2) using the ggplot2^47^ package.

To ensure each chr21 centromeric region was accurately assembled, we aligned PacBio HiFi reads generated from the sample to the relevant genome assembly using pbmm2 (v.1.1.0) (https://github.com/PacificBiosciences/pbmm2) and assessed for uniform read depth across the centromeric region with NucFreq^61^ (https://github.com/mrvollger/NucFreq). We also confirmed that the extracted centromeric contig sequences contained chr21 α-satellite HORs and not chr13 ones by comparing the sequences to the chr21 and chr13 α-satellite monomer units extracted from the T2T-CHM13 reference genome using StringDecomposer^62^ (https://github.com/ablab/stringdecomposer). Finally, we generated pairwise sequence identity heatmaps of each centromere using StainedGlass^49^ as previously described (https://github.com/mrvollger/StainedGlass).

Another 44 population chr21 centromere sizes were obtained from the recently published HGSVC data.^12^ Additionally, 115 female and 101 male samples from the HPRC year 2 release as well as 30 female and 22 male samples from the Arab pangenome reference ^11^ were processed and validated with CenMap (v.0.3.1) (https://github.com/logsdon-lab/CenMAP), a recently developed pipeline for centromere mapping and annotation, to calculate their chr21 centromere sizes. For these samples, if CenMAP identified more than two chr21 or two chr13 centromeres to any individual, the assemblies were manually inspected as described above for the families with T21. If we were not able to confidently assign the contigs to chr21 after manual inspection, the samples were excluded.

### Contig fixing and patching

Centromeric contigs that showed misassemblies or collapses during NucFreq^61^ analysis (above) were corrected by the following procedure. Sequencing reads mapping to the misassembly/collapse and flanking regions were extracted and locally assembled using the module Canu from the tool HiCanu.^63^ The local assemblies were validated using NucFreq^61^ as described previously and inserted in the original assembly.

Verkko^42^ genome assemblies containing errors were corrected with an alternative assembly generated via hifiasm^43^ or vice versa. In brief, we generated assemblies of each genome with hifiasm^43^ (v.0.19.5 or newer) and the following command: hifiasm -o {output} -t {threads} –ul {ultra-long read fastq} {hifi reads fastq}. We then aligned the hifiasm assemblies to the Verkko ones with minimap2^53^ (v.2.24) and the following command: minimap2 -t {threads} -I 15G -a –eqx -x asm20 -s 5000. We identified the corresponding locations of the centromeric regions in each assembly and then determined the α-satellite HOR array composition of each with HumAS-HMMER (https://github.com/fedorrik/HumAS-HMMER_for_AnVIL) and the following command: hmmer-run.sh {path_to_directory_with_fasta} AS-HORs-hmmer3.0-170921.hmm {threads}. We compared the α-satellite HOR compositions of each array and then replaced erroneous sequences in the Verkko assembly with the corresponding sequence in the hifiasm assembly (or vice versa). To ensure that we had resolved the assembly error, we aligned PacBio HiFi reads from the same genome to the new assembly and ran NucFreq^61^ to validate the assembly.

### Centromere alignments and dotplots

AG16777 proband and parental centromere sequences were aligned using minimap2^53^ (v.2.26) and the following command: minimap2 -cx asm5 {proband_centromeric_fasta} {corresponding_parental_centromeric_fasta} > alignment.paf. The results in.paf format were visualized with R^48^ (v.4.2.2) using the ggplot2^47^ package. Proband H3 and father H3 AluY centromeric insertions with 10 kbp padding sequence on both sides were aligned with BLAST.^64^

### CpG methylation and CDR analysis

All ONT sample data were basecalled with guppy v.6.3.7 or newer to generate BAM files containing methylation tags linked to each read. HiFi data were basecalled using CCS (v.6.4.0 or newer) (https://github.com/PacificBiosciences/ccs), Primrose (v.1.4.0 or newer), or Jasmine (v.2.0.0 or newer) (https://github.com/PacificBiosciences/jasmine), which generated the equivalent BAM files containing the methylation tags linked to each read. Jasmine 5mC predictions were used as a validation for the centromeric methylation profiles. In brief, we extracted the reads for each sample and aligned them to their respective genome assembly using Winnowmap^65^ (v.2.03 or newer) or minimap2^53^ (v.2.28). We then used modbam2bed (https://github.com/epi2me-labs/modbam2bed) or modkit (https://github.com/nanoporetech/modkit) to obtain bedMethyl files.

Using the bedMethyl files as an input and an early developer version of CDR-Finder,^66^ we identified the CDRs for each chr21 centromere. The tool first divides the region of interest (the centromeric α-satellite arrays with padding sequence on both sides, e.g., coordinates for proband H1: unassigned-0003018:0-2000000) in sequential 5-kbp bins and calculates their average methylation frequency (if a bin does not have an assigned value, the bin is excluded). It then computes the median CpG methylation frequency for each window containing α-satellite sequences (based on RepeatMasker^60^ output). It selects the bins with a lower methylation frequency than the median of the region and merges consecutive bins into candidate CDRs. These candidate CDRs are then evaluated on two criteria: whether or not they have flanking windows on either side with greater than the maximum percent methylation (defined as within 1 standard deviation of the maximum) for the entire region and if they clear a user-specified size threshold (25 kbp). Each call is annotated with a confidence label: “high confidence” if it clears both criteria or “low confidence” if it does not. The low-confidence labels are appended with either “neighbor_peaks” for the first criterion and/or “size” for the second criterion. In small centromeres where the median can be quite low due to the CDR taking up the majority of the centromeric sequence space, the initial search algorithm of taking windows below the median is flipped to identify the windows above the median and thus not part of the CDR. After removing these windows from consideration, what remains are candidate CDRs, and the algorithm continues as normal. All CDR calls were manually confirmed by inspecting the read alignments. False positives at the boundaries of the centromeres as well as calls not confirmed by the reads and due to low coverage were excluded. Centromeres other than chr21 were characterized with CenMAP (v.0.3.1) (https://github.com/logsdon-lab/CenMAP), and their methylation profile and CDR calls were calculated with the latest versions of CDR-Finder^66^ (v.1.0.1 or newer) (https://github.com/EichlerLab/CDR-Finder).

For each AG167 and 7210079 family member’s CDR, we calculated the percentage of methylated CpGs (defined as having >50% methylated CpGs at the same position across all UL-ONT reads) within the total CDR window (from start of the first CDR window to the end of the last CDR window) using the formula (number of methylated CpGs within the CDR window) × 100/(number of CpGs within the CDR window). To compare the methylation level of two CDRs, we ran a chi-squared test using the number of methylated CpGs (as defined above) and unmethylated ones (number of total CpGs minus the number of methylated CpGs). However, given the high number of observations (i.e., number of CpG sites; e.g., 6,941 on average for each proband AG1677 CDRs), the chi-squared *p* values detected even small variations as significant. Thus, we performed a Cramer’s V test to measure the effect size and have an estimate of the biological difference (Cramer’s V test <0.1, negligible; 0.1, weak; 0.3, moderate; 0.5, strong).

### SV and SNV calling

To study the total number of variants in the families with T21, we aligned all HiFi reads to T2T-CHM13 and GRCh38 using pbmm2 (v.1.13.1–0) (https://github.com/PacificBiosciences/pbmm2) with the “HiFi” preset. We first employed Somalier^67^ (v.0.2.19) to determine sample ancestry, comparing them to the samples of the 1000 Genome Project.^68^ The resulting BAM files were used to call SVs with PBSV (v.2.9.0) (https://github.com/PacificBiosciences/pbsv), Sniffles^69^ (v.2.2) (https://github.com/fritzsedlazeck/Sniffles), and Delly^70^ (v.1.2.6). Single-nucleotide variants (SNVs) and indels were called with DeepVariant^71^ (v.1.4.0). SVs were filtered to retain exclusively insertions and deletions between 50 bp and 100 kbp. SNVs were filtered using QUAL>=25 and DP>=10 filters. All variants on chrX, chrY, chr21, and chrM were excluded to correctly compare individuals with trisomies and control individuals and samples across sexes. We used the same tools to call SVs from HPRC^6^ and HGSVC^12^ samples using both references (*n* = 296; 33% AFR), and we obtained the SNV/indel data from Logsdon et al.^12^ (39 samples, 54% AFR; reference: GRCh38).

To count rare SVs and *de novo* SNVs, we only considered proband AG16777, since both parents were available for this family. We used a strategy similar to that employed in Sui et al.^72^ In brief, we called SVs aligning AG16777 phased assembly to GRCh38 and using PAV^4^ (v.2.3.4). We then retained only variants called from HiFi reads aligned to the same reference genome and supported by PBSV (v.2.9.0) (https://github.com/PacificBiosciences/pbsv), Sniffles^69^ (v.2.2) (https://github.com/fritzsedlazeck/Sniffles), and Delly^70^ (v.1.2.6). Finally, we removed common SVs found in population control individuals (*n* = 569).^49^ SNVs and indels were called from HiFi read alignments by DeepVariant^71^ (v.1.4.0) using GRCh38 as the reference for all the individuals from the families with T21. The resulting gVCFs were merged using GLnexus^73^ (v.1.4.1) (https://github.com/dnanexus-rnd/GLnexus), and variants located in repetitive regions (segmental duplications, repetitive sequences, gaps, centromeres, and homopolymer regions) were removed. We required the variants to be called without any missing genotype (GT) (i.e., “./.”). We then retained only the variants of proband AG16777 with GT quality (GQ) > 20, read depth (DP) > 10, and GT = “0/1” that were absent in the other samples (GT = 0/0 and AD_alt = 0) to exclude potential errors caused by batch effect biases. We obtained further support from ONT read alignment. For both HiFi or ONT reads, we considered a minimum base quality >10. We also required at least four ONT reads to support the *de novo* candidates, and we excluded candidates within 1 kbp from each other. Finally, we retained only the *de novo* SNVs and indels that could be confirmed by visual inspection of HiFi and ONT alignments in the Integrative Genome Viewer.^73^

### Phylogenetic analysis

For the phylogenetic reconstruction of chr21 centromere haplotypes, we first aligned a chimpanzee assembly to the T2T-CHM13 reference genome using minimap2^53^ with the following options: “-t {threads} -I 15G -x asm5 -a –secondary = no.” We identified a chr21 pericentromeric region on the q-arm where only two chimpanzee contigs mapped (chr21:11855000–11875000). The chimpanzee alignment was used as an outgroup for the analysis. We aligned all the human assemblies available against the T2T-CHM13 reference genome as described above and selected only the samples with chr21 centromeric contigs spanning the selected pericentromeric window. We then extracted the sequences mapping to the pericentromeric region of interest of each sample. Using these sequences, we generated a multi-alignment with the tool MAFFT^74^^,^^75^ (v.7.453) with the options “–auto –thread {threads}.” The maximum-likelihood phylogeny tree was constructed with IQ-TREE^76^ (v.2.1.2) with the following options: “-T {threads} -m MFB -B 1000.” Divergence time among haplotypes was estimated with IQ-TREE^76^ (options: “–keep-ident –date-tip 0 –date-ci 100 -redo”) using the MAFFT multi-alignment, the consensus tree, and a time estimate file containing the known divergence time between human and chimpanzee (6.4 million years) as inputs. The resulting tree was visualized with iTOL^77^ and FigTree (https://github.com/rambaut/figtree).

## Results

### Characterization of all three chr21 centromeres in an individual with T21

**Figure 1:**
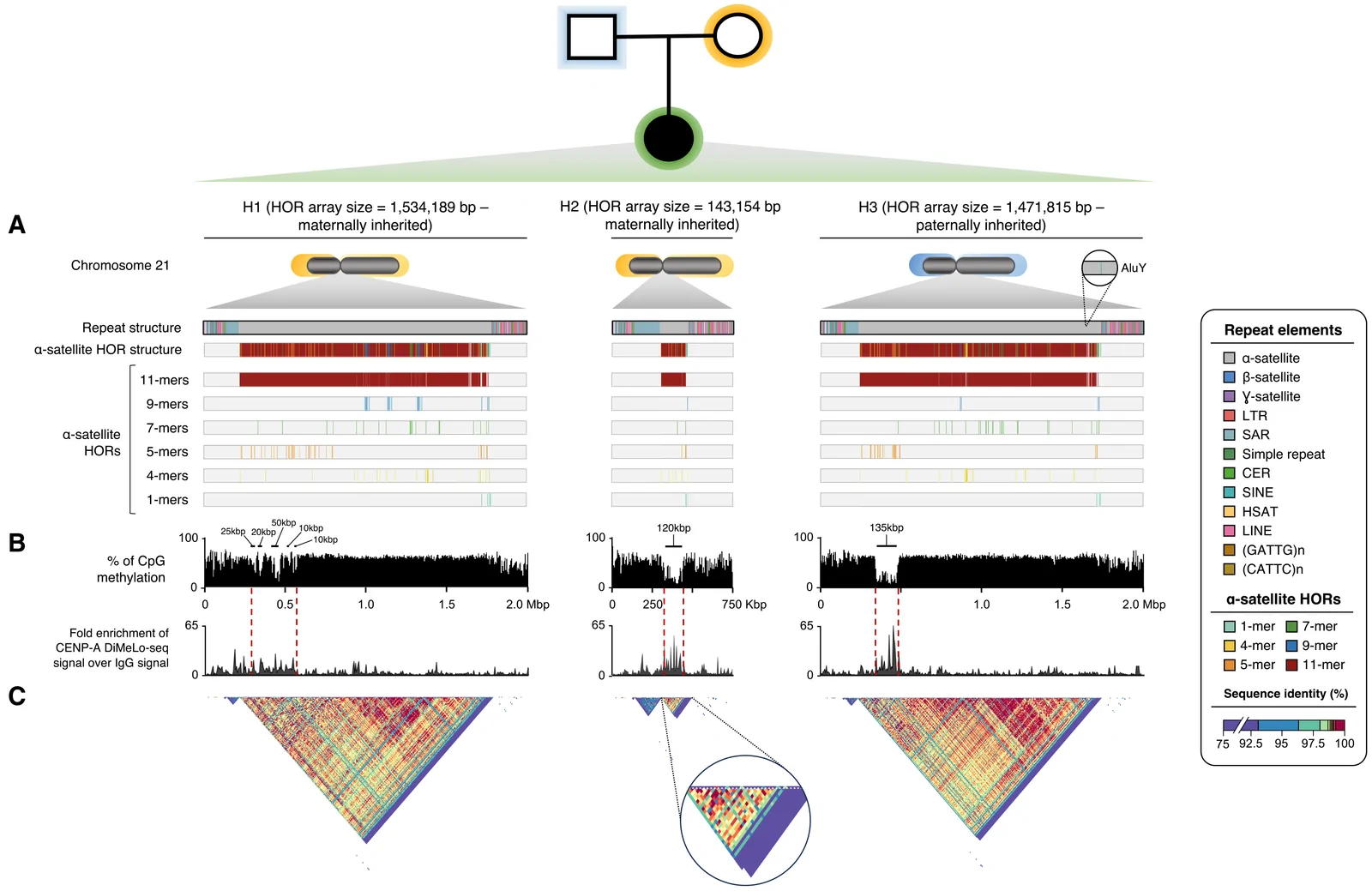
Genetic composition and epigenetic profile of three chromosome 21 centromeres from an individual with trisomy 21 (A) Size and repeat composition of three centromeric haplotypes for an individual with trisomy 21 (T21) (Coriell ID: AG16777). Different α-satellite higher-order repeat (HOR) monomers mapping within the α-satellite array are colored (see legend). Only α-satellite HOR monomers present more than ten times in at least one haplotype are shown (see Table S1 for complete monomer composition). (B) Methylation profile and fold enrichment of CENP-A DiMeLo-seq signal over IgG signal, visualized with 5-kbp bins. (C) Pairwise sequence identity heatmaps (StainedGlass^49^). Sequence identity is depicted in 5-kbp windows across the centromeres, revealing regions of high- and low-identity homology within each α-satellite HOR array (see legend).

To study the chr21 centromeres in individuals with T21, we initially sequenced the DNA of a proband (AG16777) (maternal age at childbirth, 29 years) using both PacBio HiFi sequence data (59×) and UL-ONT sequence data (23×). We used Verkko, a hybrid genome assembly workflow,^42^ to assemble all proband chr21 centromere haplotypes (total genome assembly size = 6.118 Gbp, number of contigs = 3,466, contig N50 = 7.72 Mbp). After standard validation and quality control^15^^,^^17^^,^^61^ (material and methods and Figure S1), sequence assembly of phased chr21 centromere haplotypes confirmed an MMIE, where haplotypes 1 (H1) and 2 (H2) arose from maternal nondisjunction, and haplotype 3 (H3) was inherited from the father (Figure 1A). We note that this proband carries a mosaic translocation between the long arms of chr21 and chr22 in more than half of the cells examined by the Coriell Institute for Medical Research biobank, which is absent in both mother and father. We independently validated this mosaicism by Strand-seq and estimated it to represent 12.5% of the cells (Figure S2; material and methods; supplemental notes). Being somatic, this translocation likely occurred postzygotically after the genesis of T21, which is instead germline and does not involve the centromeres. Therefore, it is unlikely to affect our analysis.

The proband’s H1 and H3 centromeres are the most similar in size, with α-satellite HOR arrays of ∼1.53 Mbp and ∼1.47 Mbp, respectively. By contrast, the maternal H2 centromere is 10.7-fold smaller, with an α-satellite HOR array of only 143 kbp (Figure 1A). The α-satellite HOR array sequences from all three haplotypes are predominantly composed of 11-mer α-satellite HORs (∼86%–93%), with a smattering of rarer α-satellite HORs 2-, 10-, 13-, 17-, and 18-mers in length (each constituting <3% of the sequence) (Figure 1A and Table S1). Other than their size, the three chr21 centromeres share a remarkably similar organization, with the α-satellite HOR array flanked by human satellite 1a (HSat1a; annotated as “SAR’’ by RepeatMasker) on the p-arm and various classes of mobile elements (short and long interspersed elements [SINEs and LINEs]) on the q-arm. Also, H3 is distinguished by a 317-bp AluY retrotransposon insertion in its α-satellite HOR array (Figure 1A).

Given the use of long-read sequencing, which allows for CpG methylation to be detected, we were able to assess the position and distribution of each chr21 CDR, the pocket of hypomethylated DNA indicating the location of the kinetochore.^15^^,^^16^^,^^26^ Both H2 and H3 match this description with a CDR of 120 kbp and 135 kbp, respectively. By contrast, the maternally inherited H1 is fragmented into five smaller pockets ranging from 10 to 50 kbp and spanning across 280 kbp of centromeric α-satellite HORs (Figure 1B). We quantified the level of methylation of each CDR (Table S2 and supplemental notes) using ONT sequencing data and orthogonally validated the centromere methylation profiles using PacBio HiFi sequencing data (Figure S3).

The CDR is considered a proxy for kinetochore attachment and coincides with the location of the centromeric proteins CENP-A and CENP-C. Thus, we also assessed the level of CENP-A and CENP-C on chr21 centromeres in proband AG16777 and their parents using immunofluorescence combined with FISH (IF-FISH), targeting the α-satellite sequence and the two centromeric proteins (Figure S4). Second, we performed CENP-A DiMeLo-seq, a single-molecule approach that couples the specificity of CENP-A protein-antibody recognition with long-read sequencing to improve sensitivity of mapping CENP-A within α-satellite DNA^16^ (Figure 1B). IF-FISH did not show any statistically significant difference between CENP-A and CENP-C (Figure S4), and similarly, CENP-A measurements via DiMeLo-seq showed CENP-A accumulation over each haplotype (supplemental notes). This suggests no significant epigenetic differences among the centromere haplotypes.

For each chr21 centromere, we also generated sequence identity heatmaps, based on pairwise sequence alignment of 5-kbp segments using StainedGlass^49^ (Figure 1C). All three haplotypes show common compositional features, with the most identical segments mapping to the central section of the α-satellite HOR array but not corresponding to the CDR, with the exception of H2, due to its smaller size. This structure is also similar to the complete chr21 α-satellite HOR arrays described for two complete hydatidiform moles (CHM1 and CHM13)^16^^,^^17^ as well as population samples ^12^ and generally follows the Smith organizational model^78^ with monomeric α-satellite at the edges. For both H1 and H3 centromeres, the CDR and the predicted site of kinetochore attachment occur peripherally in a region composed of more heterogeneous α-satellite HOR cassettes (with an admixture of 5- and 11-mer HORs). Of note, we also characterized additional proband centromeres from 13 other chromosomes for this family (Figure S5). For chromosomes 4, 12, and 15, both homologous centromeres were assembled without errors. We did not identify other centromeres exhibiting an extreme difference in size between homologs, with the largest detected being between chr15 homologs (∼2-fold difference).

### Intergenerational transmission of chr21 centromeres

To understand the inheritance of the chr21 centromeres, we next sequenced and assembled the genomes of both parents using DNA extracted from lymphoblastoid cell lines and fully reconstructed each of the centromere haplotypes from the diploid parental cell lines (mother: AG16778; father: AG16782). We generated PacBio HiFi sequencing data at 37× coverage for the mother and 40× for the father and complemented these data with UL-ONT data at 25× and 12× coverage, respectively, to generate *de novo* assemblies (mother: total genome assembly size = 6.078 Gbp, number of contigs = 4,827, contig N50 = 5.41 Mbp; father: total genome assembly size = 5.947 Gbp, number of contigs = 5,032, contig N50 = 5.01 Mbp). Centromeric contigs from chr21 were extracted, validated, and compared to those of the child with T21 (see material and methods for assembly correction). No additional complete and correctly assembled centromeres were found in the maternal assembly, likely due to the lower coverage of long-read sequencing data.

**Figure 2:**
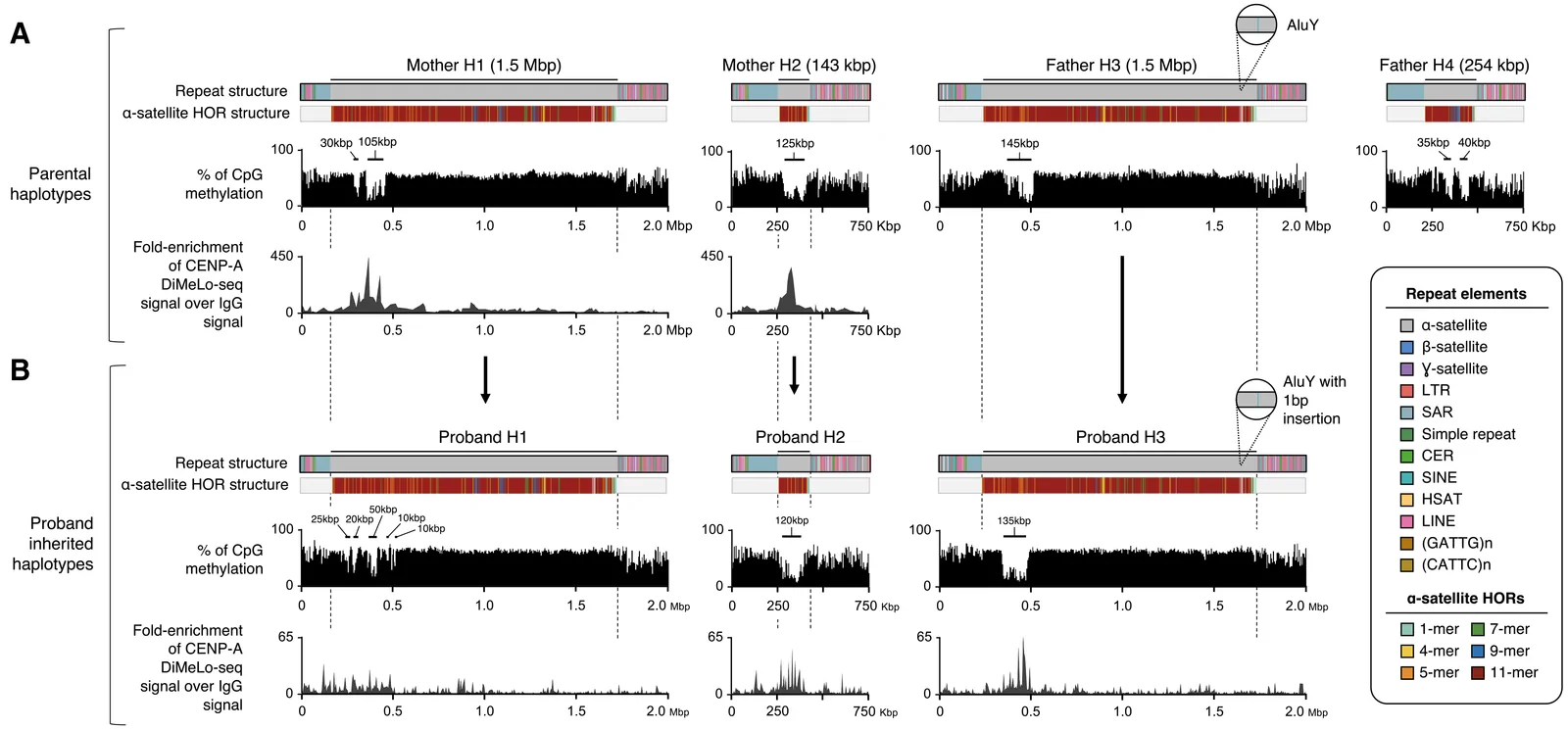
Parent-proband transmission of chromosome 21 centromeres Composition and organization of α-satellite HOR arrays (only α-satellite HORs present more than ten times in at least one haplotype are shown; see Table S1 for complete HOR composition), with CpG methylation profiles of (A) parental chromosome 21 (chr21) centromeres and (B) transmitted proband chr21 centromeres.

Sequencing confirmed that the largest and smallest chr21 centromeres (H1 and H2, respectively) were maternally transmitted, while H3 originated from the father (Figures 2A and 2B). Sequence identity between parent and proband haplotypes was >99.99% identical, confirming the accuracy of the assembly, the stability of the centromeres intergenerationally and in cell culture (Figure S6), and lack of recombination inside the centromeric α-satellite HOR array. No difference in the α-satellite repeat composition, organization, or array size was observed, except for a 1-bp deletion in the poly(T) tract associated with an AluY insertion on the paternal haplotype (H3) when compared to the proband (Figure S7). We note that the second non-transmitted paternal haplotype (father H4) has an α-satellite HOR array size of 254 kbp (Figure 2A). This is 112 kbp larger than the small maternal haplotype (H2). Like the other haplotypes, the 11-mer α-satellite HORs constitute the majority of its array sequence (∼86%), with 2- to 5-, 7-, 9-, 10-, and 13-mer HORs at lower frequency (each composing <6% of the array sequence).

Comparing the centromeric CpG methylation profiles between the parents and the proband, we find that the methylation pattern observed in proband H1 (five hypomethylated pockets 10–50 kbp in size) appears to be specific to the proband (Figures 2A and 2B; supplemental notes; see Figure S3 for orthogonal validation). The mother shows a more typical CpG methylation profile, with two clear CDRs of 30 and 105 kbp in length. The CDRs of chr21 H2 and H3 centromeres differ by only an estimated 5–10 kbp in size between generations.

### Analysis of chr21 centromeres in additional families with Down syndrome

After the analysis of the index trio, we generated PacBio HiFi and UL-ONT sequence data from seven additional families: six mother-proband duos where MMIEs had been previously identified and one singleton where parental DNA was not available. Importantly, unlike the original trio, in six of these families both blood and cell-line DNA material were available for investigation. Because the maternal age of the index case was 29 years, we specifically selected mothers representing a young age range (20–34 years) (Table S3). Despite the well-known maternal age effect with T21, we note, however, that this age range still represents the majority of the cases in the extended collection from the Atlanta and National Down Syndrome Projects^79^^,^^80^ (maternal age at birth < 35 for ∼54% of the cases; 2,171/3,985), with a mean maternal age at birth of 32.9 years old. We assembled their genomes with the same methods used for the original family (probands’ mean genome assembly size = 6.072 Gbp, number of contigs = 1,237, contig N50 = 45.84 Mbp; mothers’ mean genome assembly size = 6.064 Gbp, number of contigs = 2,217, contig N50 = 35.16 Mbp) and analyzed their chr21 centromeric sequences. Of the proband centromeres, 95% (20/21) were completely assembled, while 67% (8/12) of the maternal centromeric haplotypes were assembled without any detectable error. As seen in the index trio, the maternal centromeres were transmitted intergenerationally without any evidence of recombination within the α-satellite HOR array.

**Figure 3:**
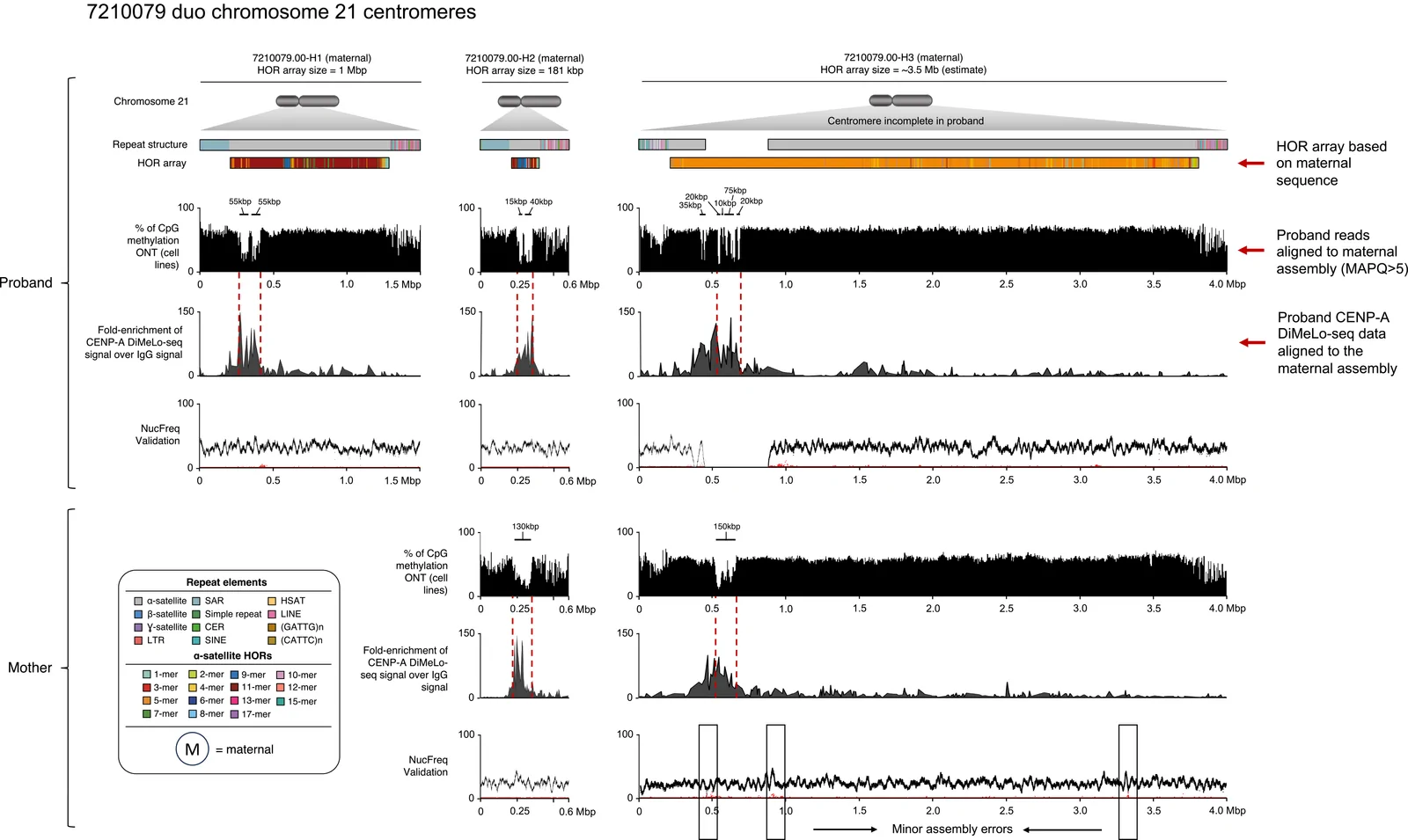
Chromosome 21 centromeres in duo 7210079 Repeat structure, α-satellite HOR array organization, CpG methylation profile, fold enrichment of CENP-A DiMeLo-seq signal over IgG signal visualized with 5-kbp bins, and NucFreq validation of each chr21 centromere are shown for the 7210079 mother-child duo. The 7210079.00 proband H3 was not completely assembled. Since the same haplotype was assembled in the mother, we used the maternal assembly as a reference to study the methylation profile in the proband.

In this extended cohort, we identified one additional family showing extreme centromere size differences as described in the original trio with T21. Duo 7210079 shows a 19.4-fold chr21 centromere size difference between maternal homologs (original trio: 10.7-fold) with a small centromere α-satellite HOR array of only 181 kbp (original trio: 143 kbp) and a longer one of ∼3.5 Mbp (original trio: 1.5 Mbp) (Figure 3). We note that mother 7210079.20 had an age at childbirth (27 years) comparable to the original trio mother AG16778 (29 years). Since the 7210079.00-H3 centromere was not assembled completely in the proband, we leveraged the maternal locus (assembled contiguously with few sequence errors) as a reference to align UL-ONT reads both from mother and child and compare methylation profiles and CDR structure. Specifically, the maternal CDR maps to a unique window of 150 kbp more proximally toward the p-arm, while in the proband, the CDR is divided into five smaller windows (size range, 75–10 kbp) despite remaining in the same genomic area (Figure 3). IF-FISH targeting CENP-A and CENP-C (Figure S4) and CENP-A DiMeLo-seq signals (Figure 3) showed accumulation of both proteins on each maternally inherited haplotype in the proband and mother, similarly to what was observed in the index trio (supplemental notes).

We also characterized 35 additional correctly assembled centromeres in duo 7210079 (17 in proband, 18 in mother) (Figure S5). In proband 7210079.00, we were able to study both haplotypes for chr2, chr6, chr8, chr15, and chr22, and for the mother, chr11, chr13, and chr16. We did not detect any other asymmetric centromeres, indicating that centromere asymmetry for these two families is a feature of chr21, which was similar to our analysis of AG16777. Among the remaining probands (Figure S8), all have maternal homologs that are at least 1 Mbp large and a size difference of 2-fold or less (Table S3).

With respect to sequence composition, most proband centromeric α-satellite HOR arrays in this cohort are similar to the original, composed predominantly of 11-mer α-satellite HORs (∼77%–94%), with rarer HORs representing the rest of the sequence. Notably, 4710291.00-H3 has 10% of its sequence composed of a 4-mer α-satellite HOR, 7210239.00-H1 has 8%, and 7210008.00-H1 has 5%. The smallest haplotype of this cohort, 7210079.00-H1, has 9% of its sequence organized in a 9-mer HOR (Table S1). However, 7210079.00-H3 is unique compared to all the other centromeres. Based on the maternal α-satellite HOR array structure (Figure 3), it is the largest centromere sequenced among all characterized (α-satellite HOR array of ∼3.5 Mbp), with almost 1 Mbp more sequence than the second largest (4710291.00-H3). Also, its predominant α-satellite HOR array unit is a 5-mer (88%), with only 6.65% of its sequence made up of the more typical 11-mer units. Overall, none of these genomes had an increased number of variants compared to the control individuals, nor did the index trio data show a higher mutational burden in the proband (Figure S9; material and methods; supplemental notes).

Finally, we attempted to control for cell line versus primary tissue differences with respect to the CDR. Because both blood and cell-line DNA were available for a subset of the probands, we compared the chr21 centromere methylation patterns for the two sample types for the five probands with complete centromeres. PacBio HiFi data were generated from blood DNA, and UL-ONT data were generated from cell-line DNA. We confirm that the CDR positions remain the same from primary tissue (blood) to cell line (lymphoblastoid) while the intensity of the methylation dips appears to differ (Figure S10). Specifically, we observe an overall higher level of methylation in blood and more readily defined CDR in lymphoblastoid cell lines (Figure S10). This was concluded by observing ONT and PacBio HiFi data processed with the latest methylation callers dorado (v.0.4.2) or guppy (v.6.3.7 or newer) and Jasmine (v.2.2.1), respectively. We also confirmed the methylation callers’ concordance in the original trio (Figure S3) and other samples (Figure S10), for which the two long-read datasets were generated from cell lines.

### Population chr21 centromere diversity and phylogeny

**Figure 4:**
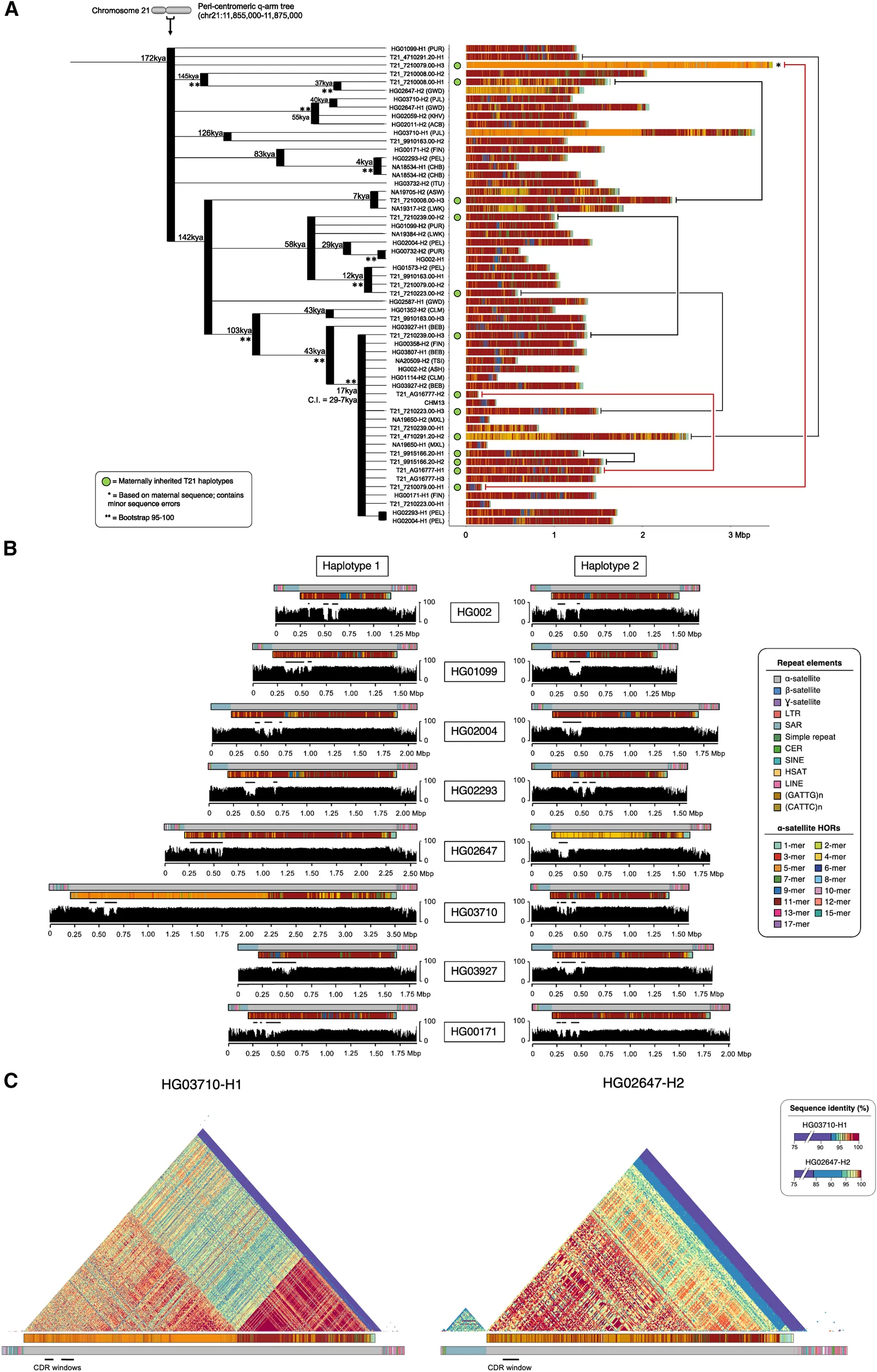
Diversity in α-satellite HOR array length, composition, and CpG methylation profile among diverse chr21 centromere haplotypes (A) Maximum-likelihood phylogenetic tree based on a multiple sequence alignment of a 20-kbp segment from the pericentromeric q-arm regions from 57 unique completely sequenced chr21 centromeres. The HOR array structure of each is depicted, and estimated divergence time for each cluster is projected onto the nodes. Bootstrap values 95–100 are marked with asterisks (^∗∗^). Black lines connect homologous chr21 centromeres found in mothers, which were both transmitted to their child with T21. Red lines indicate the homologs exhibiting extreme centromere size asymmetry. (B) CpG methylation profiles of 16 population haplotypes (see also Figure S11). (C) Pairwise sequence identity heatmaps (generated with StainedGlass^49^ using 5-kbp windows) across chr21 centromeres enriched with 5-mer and 4-mer α-satellite HORs (see legend).

To understand the genetic and epigenetic diversity of chromosome 21 centromeres in the general population, we initially selected 35 fully resolved chr21 centromere haplotypes from 16 different ethnic groups representing all five ancestry superpopulations as defined by the 1000 Genomes Project.^68^ We observed considerable diversity in α-satellite HOR array composition and length among these 35 complete chr21 centromeres. While the most prevalent α-satellite HOR is 11-mers in length and comprises ∼83%–∼95% of the α-satellite HOR array sequence, in most of the complete chr21 centromeres, we also detect less abundant α-satellite HORs that are 4, 5, 12, 14, 15, 16, and 20 α-satellite monomers in length (Table S1; Figures 4A and 4B). Some haplotypes among individuals of African ancestry (3 out of 7 centromeres) have 5% or more of their sequence composed of 4-mer α-satellite HORs (∼5% to ∼15%). Similarly, both probands of African descent from our families with T21 have one haplotype (2 out of 4) showing the same feature (∼5% and ∼10% of 4-mer) as well as another centromere (8%) in a proband of European ancestry (Table S1). These data, when aggregated, suggest that individuals of African descent harbor a higher amount of centromeric sequence organized as 4-mer α-satellite HOR (Fisher’s exact test *p* value = 0.001).

We note two chr21 centromere haplotypes whose composition differs markedly from the predominant 11-mer α-satellite HOR organization: an individual of Gambian (GWD) descent, HG02647, carries a 4-mer α-satellite HOR comprising ∼15% of its H2 centromere sequence; and an individual of Punjabi (PJB) descent, HG03710, with 44% of its H1 centromere sequence composed of 5-mer α-satellite HORs (Figure 4C and Table S1). The 7210079.00-H3 described earlier is the only other chr21 centromere containing a majority of 5-mer HORs (88%) and, along with HG03710-H1, they represent the two largest chr21 centromeres described in this analysis. Sequence identity plots (Figure 4C) also reveal that the 11-mer-enriched α-satellite HOR region of HG03710-H1 shows greater sequence identity when compared to the 5-mer-enriched regions. In contrast, the 4-mer α-satellite HOR of HG02647-H2 shows greater sequence identity when compared to other flanking monomers (Figure 4C). If sequence divergence is a proxy for evolutionary age, our analyses may suggest that the 4-mer α-satellite HOR has more recently evolved, while the 5-mer α-satellite HOR is more ancient. Notwithstanding, both of these rarer centromere haplotypes map their CDRs to the noncanonical 5-mer and 4-mer α-satellite HORs instead of the 11-mer.

We also compared the diversity in length and location of the CDRs for the various chr21 centromere haplotypes. The number of CDRs typically range from one to four and span a total of 90–335 kbp in total length (Figure 4B and Figure S11). Some haplotypes, such as HG02647-H1 and HG03927-H1, show large CDR windows with peaks of higher methylation within. We observe four chr21 centromere haplotypes that have two hypomethylated regions separated by more than 100 kbp: HG002-H1 and H2; HG02293-H1; and HG01573-H2. The dispersed CDRs tend to be smaller (10–25 kbp) and contained within a single pocket, except for HG01573-H2, which has two. Irrespective of sequence composition, 91% (20/22) of the chr21 CDRs map to the p-arm of the centromere, suggesting a polarity or preference for CDR location (Figure S11).

Finally, we constructed a chr21 centromere phylogenetic tree using 20 kbp of flanking pericentromeric sequence in the q-arm from the genomes assembled in this study and chimpanzee as an outgroup (material and methods). Pericentromeric DNA has the advantage that it is unique; thus, it can be orthogonally aligned between individuals and species without the excessive turnover or multi-mapping associated with α-satellite HORs. More importantly, the absence of centromere recombination means that it is in near-complete linkage disequilibrium with the α-satellite HOR array, as previously demonstrated,^17^ by showing that pericentromeric p- and q-arm trees located ∼2 Mbp apart have nearly identical topology. Therefore, we can use the highly accurate and unique assembly as a proxy to reconstruct the evolutionary history and coalescence of any centromere and, in this case, chr21.

We find that 50% (11/22) of T21 haplotypes included in the analysis are part of a large monophyletic clade comprising 40% (23/57) of all chr21 centromeres (Figure 4A). The clade harbors the largest (H1) and smallest (H2) centromeres for proband AG16777, which are predicted to have arisen from a common ancestor very recently. We estimate they diverged 17 thousand years ago (kya), despite the fact that these two haplotypes vary by more than 10-fold in their overall length. It also contains the small centromere found in the second family (7910079) where extreme centromere size asymmetry was observed. We note that this particular clade is significantly enriched for centromeres smaller than 500 kbp (Fisher’s exact test *p* value = 0.001). In contrast, our analysis also reveals that the two large chr21 centromeres containing an abundance of 5-mer α-satellite HORs (7210079.00-H3 and HG03710-H1) diverged much earlier (126 and 172 kya) compared to the other haplotypes.

### Asymmetry of centromeric α-satellite HOR length

To establish a baseline for centromere length distributions in the human population, we compared the T21 centromeres to an expanded set of 287 completely assembled chr21 centromeres from control individuals. The control cohort consists of two hydatidiform mole (CHM13 and CHM1) chr21 centromeres, 26 chr21 centromeres (from 13 diploid samples) from the Arab pangenome reference project,^11^ 184 chr21 centromeres (from 92 diploid samples) from the recently released genomes part of the HPRC (year 2 release),^6^ 40 chr21 centromeres recently published by the HGSVC,^4^^,^^12^ and the additional 35 centromeres from the two consortia described in the previous paragraph (Table S4).

**Figure 5:**
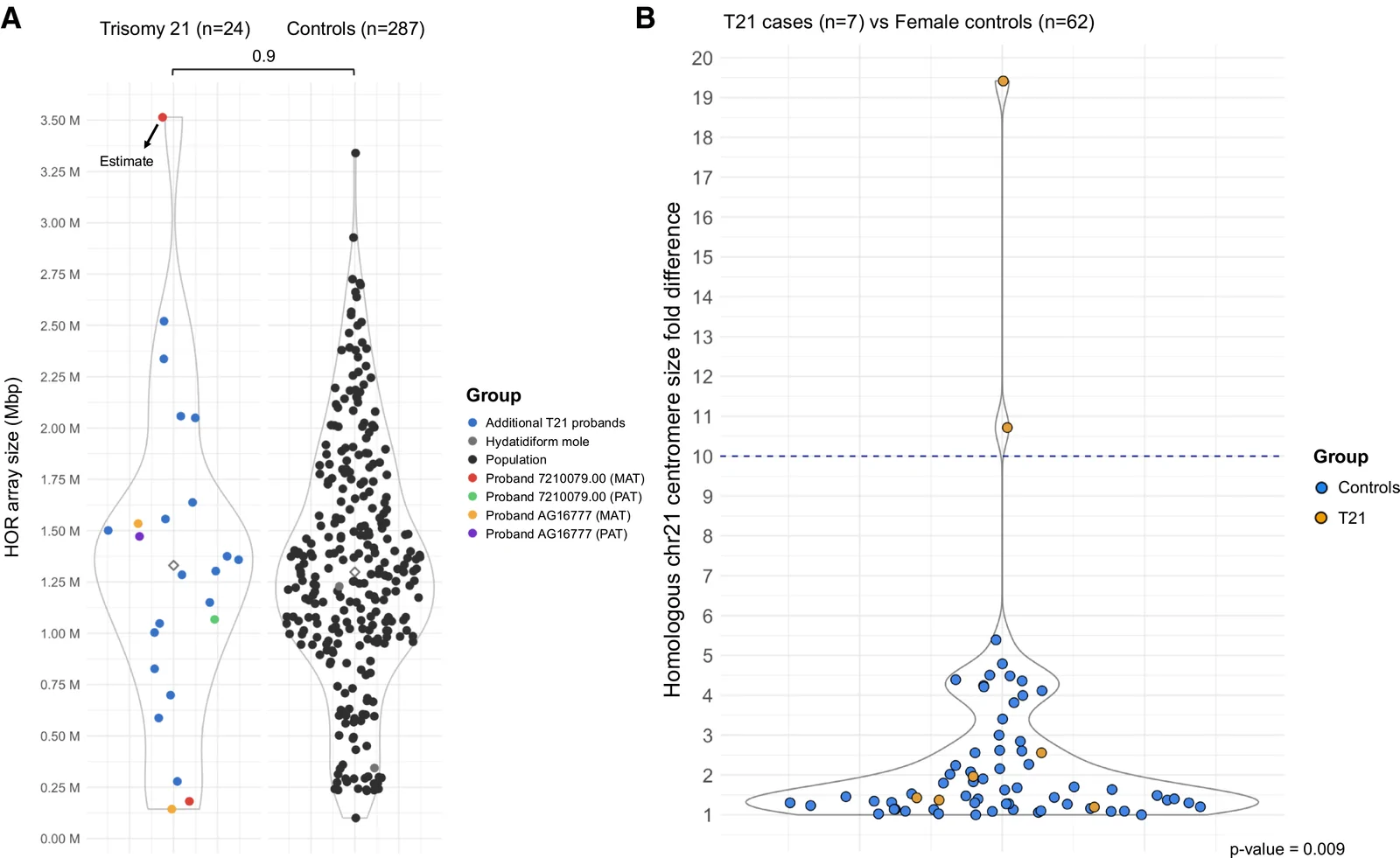
Comparison between centromere HOR array sizes from individuals with T21 and control individuals (A) Size-distribution comparison of chr21 centromeric α-satellite HOR arrays from individuals with T21 and population samples (Welch's two samples *t* test *p* value = 0.9). (B) Homologous chr21 centromere size fold differences in individuals with T21 (only maternally inherited centromeres; orange) and female population samples (blue). Fisher's exact test *p* value (0.009) indicates a significant difference in extreme asymmetry cases between the two groups.

Comparing the size distribution of all control haplotypes (*n* = 287) to those from individuals with T21 (*n* = 24), we find no statistically significant difference in length (Welch’s two sample *t* test *p* value = 0.9) (Figure 5A). Similarly, the frequency of small centromeres (<500 kbp) also showed no significant difference between the two groups (3/24 in T21 and 27/287 in the general population groups; Fisher’s exact test *p* value = 0.72), despite an earlier report suggesting the contrary.^38^ Notwithstanding, the small centromeres identified in AG167 and 7210079 families (mothers and children) remain among the smallest chr21 α-satellite HOR arrays sequenced in female individuals, measuring 143 kbp and 181 kbp, respectively (Figure 5A). For the male individuals, only one centromere was found to have a smaller α-satellite HOR array, namely HG00738-H1, which represents, so far, the only chr21 centromere with an array of 100 kbp.

Given this observation, we considered the diplotype of the transmitting mothers and assayed the frequency of extreme centromere size asymmetry (>10-fold) in the population using 129 control samples (62 females and 67 males) and seven individuals with T21 for which both centromere haplotypes were fully resolved (the singleton with T21 was excluded, since we could not phase the centromeres nor determine if the centromere size remained the same between the two generations). A significant difference in extreme homologous chr21 centromere size asymmetry cases was observed between mothers with children with T21 and the general population (Figure S12) (Fisher’s exact test *p* value = 0.002). We identified two cases in the cohort of individuals with T21 where homologous centromere α-satellite HOR array length differed more than 10-fold and none in 129 control individuals. The maximum difference identified between all chr21 HOR arrays in control individuals was 5.4-fold, and in another recent population survey it was even lower (i.e., <3.5-fold),^51^ with no extreme centromere size asymmetry observed.

Considering the fundamental differences in male and female meiosis, we repeated the analysis while restricting it further to only female individuals from the general population. The number of asymmetrical centromeres remained statistically significant for families with T21 (Fisher’s exact test *p* value = 0.009) (Figure 5B). We further expanded the control cohort by including another 23 female and 22 male individuals from the HPRC year 2 release that were completely assembled but had small errors in their sequences (e.g., collapses and misjoins). In these samples, the centromere size estimate is not as accurate, but we do not expect a great difference between the estimate and their actual sizes. Once again, none exhibited extreme centromere asymmetry. If we consider these additional female samples in our comparison, the difference becomes even more statistically significant (Fisher’s exact test *p* value = 0.005). Despite being statistically significant, the pangenome is not sufficiently deep enough, nor are the number of families with T21 assessed sufficiently large enough, to conduct ancestry-matched simulations by population. Thus, while intriguing, these results should be regarded with caution until larger sets of individuals and control counterparts are examined.

## Discussion

Down syndrome was first described in 1866,^81^ and its typical T21 karyotype description was reported in 1959.^82^ Despite extensive research over the last 65 years, it is not known whether there is any genetic predisposition that makes chr21 or individual families prone to free T21. To this day, we still do not have a method to assess predisposition to this condition, nor are we aware of any direct genetic risk factor that leads to it. We could only test directly for T21 during pregnancies with invasive procedures, such as amniocentesis, until more recent, safer noninvasive prenatal tests emerged.^83^ Understanding the genetic architecture of T21 and genetic factors that predispose to it may be another important consideration in its prevention.

In this study, we present the complete sequence and epigenetic characterization of chr21 centromeres of eight individuals with free T21, seven of their mothers, and one father. In all families, T21 was caused by MMIEs, and all mothers were <35 years old at childbirth. We initially aimed to investigate chr21 centromere variability in individuals with T21 and test the hypothesis that individuals with the syndrome carry small centromeres. Previous studies postulated that “centromere strength” could be associated with the amount of centromeric DNA sequence.^31^^,^^32^^,^^33^^,^^34^ Other reports revealed a statistically significant higher number of small chr21 α-satellite HOR arrays in individuals with the syndrome compared to control individuals,^38^ suggested an association of small chr21 α-satellite HOR arrays and MMIEs,^84^ and proposed that individuals with T21 generally harbor small chr21 centromeres.^38^ Additionally, a 2003 study explored the relationship between the size of α-satellite HOR arrays on chr21 centromeres and repressed recombination, one of the known phenomena that correlates with nondisjunction, and concluded that the features are independent.^85^

In our investigation, chr21 centromeres showed remarkable variability in length, α-satellite HOR array structure, and CDR patterns within the human population (Figure 4), suggesting that chr21 is among the top four most variable centromeres in the human genome.^17^ We observed a population difference in α-satellite HOR composition, with a 4-mer α-satellite HOR being more frequent in centromeres from individuals of African ancestry (both from the general population and individuals with T21; Fisher’s exact test *p* value = 0.001). Moreover, we noticed that the largest chr21 centromeres identified (∼3.5 Mbp and ∼3.3 Mbp) are the only ones to have a large portion of their sequence composed of 5-mers (44% and 88%). We hypothesize that this 5-mer α-satellite HOR either originated from archaic hominin introgression,^51^ which would be consistent with its deeper coalescence (126 and 172 kya), or it is the result of incomplete lineage sorting. Moreover, given the sharp transition between 5- and 11-mer α-satellite HORs in HG3710-H1, this centromere could be the result of a rare occurrence of centromere recombination (Figure 4).

We show that small centromeres are found at low frequency in individuals with T21 (3/24; three centromeres smaller than 500 kbp in three individuals, two of which of maternal origin) and that T21 centromere size distribution is comparable to that of the general population (Figure 5A). Despite being present at low frequency in the general population (9%), our data do not support the observation that small centromeres alone constitute a risk factor for T21 nondisjunction. Instead, we find evidence that a fraction of the families with T21 (two of seven mothers) show extreme chr21 centromeric α-satellite HOR array size asymmetry. Small centromeres might have been previously reported in children with T21, as they make size asymmetry more likely than larger centromeres. Our results support other observations,^86^ which noted that small centromeres are not exclusive of individuals with T21; contrarily, extreme homologous chr21 centromere size asymmetry (>10-fold) appears to be absent, at least to date, in individuals without the syndrome (Figures 5B and S12). We report two cases in families (∼30%) that include an individual with T21 with similar features but none in the general population, at least based on existing population genetic diversity that has been sampled.

Our phylogenetic analysis may help explain why chr21 is so susceptible to asymmetry (Figure 4A). We estimate that rapid changes of chr21 centromere size have occurred very recently in human evolution (∼17 kya), confirmed by the fact that human chr21 centromeres are smaller than the corresponding homologs in chimpanzee and gorilla.^20^ This recent contraction in the human lineage likely facilitated such centromere size asymmetry. One possibility may be that an extreme difference between chr21 centromere sizes makes homologous alignment and pairing less efficient, complicating meiotic synapsis formation across the entire chromosome length. There is evidence demonstrating that larger domains of homology are essential for synapsis formation and/or recombination in complex organisms such as *C*. *elegans*, *Drosophila*, and mice but not in simpler organisms,^87^^,^^88^^,^^89^^,^^90^^,^^91^ which might explain some of the biological difference observed in centromere yeast studies. Recent data have also shown that the rapidly mutating short arms of the human acrocentric chromosomes are depleted for allelic recombination,^23^ possibly due to the high divergence between homologs for acrocentric short arms. Thus, centromere asymmetry might contribute to even less optimal pairing and synapsis formation on the allelic long arms, leading to precocious separation of the homologs and consequent nondisjunction. If so, it would only account for a fraction of T21 cases, as the majority (five of seven) of mothers showed no evidence of asymmetry. While these findings are intriguing, a larger number of affected individuals and control counterparts as well as experimental data are needed to test if and how asymmetry impacts meiosis, recombination, and chromosome segregation.

We also investigated, as a corollary to the genetic characterization of T21 centromere asymmetry, the number of centromeric proteins forming the kinetochore complex on each of the chr21 centromeres of family AG167 and duo 7210079. IF-FISH and DiMeLo-seq data demonstrate that none of the chr21 centromeres in mothers exhibit a lack of signal for CENP-A or CENP-C, indicating that their kinetochore function is not impaired, but we could not study the CpG methylation profile and the kinetochore protein accumulation in the maternal germ cells. Also, we could not establish the significance of the CDR changes across generations, since drastic differences have been observed in the general population as well.^13^^,^^23^ Overall, we do not have evidence that the condition arose from a pre-existing kinetochore defect in the mothers, reinforcing the hypothesis that centromere asymmetry might be a risk factor for nondisjunction but only for a minority of families.

The second cohort of families with T21 also allowed us to compare CDR differences between cell lines and a primary tissue such as blood. Little is known regarding the stability of CDRs somatically; however, our limited data on multiple samples demonstrate that CDR position between blood and cell lines remain positionally conserved. The only detected difference is a generally higher level of methylation in the blood. This could be due to multiple reasons. First, comparison of methylation callers (Figure S10) on cell-line data indicates that PacBio basecallers (Jasmine and especially the older Primrose) detect a slightly higher level of methylation. Also, HiFi reads might be mapping less precisely than the much longer UL-ONT reads. Lastly, it is possible that some highly specialized cells in the blood, which are generated from the progenitors in the bone marrow and do not typically undergo mitosis,^92^ might lose epigenetic signatures over time like those associated with CDRs.

There are several limitations to this study. First and foremost, the observation of asymmetry rests on relatively few observations, in part because the sequence and assembly of centromeres is still a costly and time-consuming endeavor. Moreover, we focused here on individuals with T21 conceived by mothers of a young age range (20–34 years) despite the known, strong association of nondisjunction with advanced maternal age. It will be important to pull apart these confounders by assessing centromere asymmetry in even younger (age <20) and older (age >34) mothers and incorporating the maternal age effect. Larger T21 population-based studies are, therefore, required to confirm this finding as well as evaluating families with MMIEs and MMIIEs. Large cohorts with short-read sequence data, such as the Atlanta or the National Down Syndrome Projects,^79^^,^^80^ have been assembled, and imputation of chr21 centromere length in these cohorts once a large enough pangenome has been amassed may be possible instead of direct sequencing. Finally, future studies should evaluate not only centromere sequence and methylation using long-read sequencing data but also the composition and location of the kinetochore complex as it relates to epigenetic differences of the CDR.

## Data and code availability

Sequencing data were submitted to the database of Genotypes and Phenotypes (dbGaP) for general research use. The accession number for the sequencing data reported in this paper is dbGaP: phs003761.v1.p1. The code used is referenced in the material and methods section.

## Acknowledgments

We thank Tonia Brown for the editing and preparation of the manuscript. This work was supported, in part, by US National Institutes of Health (NIH) grants R01HG010169 and U24HG007497 (to E.E.E.) and R00GM147352 (to G.A.L.). The content is solely the responsibility of the authors and does not necessarily represent the official views of the NIH. E.E.E. is an investigator of the 10.13039/100000011Howard Hughes Medical Institute (HHMI). The procedures followed were in accordance with the ethical standards of the responsible committee on human experimentation (institutional and national), and proper informed consent was obtained.

This article is subject to HHMI’s Open Access to Publications policy. HHMI lab heads have previously granted a nonexclusive CC BY 4.0 license to the public and a sublicensable license to HHMI in their research articles. Pursuant to those licenses, the author-accepted manuscript of this article can be made freely available under a CC BY 4.0 license immediately upon publication.

## Author contributions

F.K.M., G.A.L., and E.E.E. conceptualized the project; S.L.S., E.G.A., T.C.R., and W.H. provided the samples; J.K., G.H.G., M.A., K.M.M., and K.H. generated the long-read sequencing data; S.-C.C., K.K.O., and G.A.L. generated the DiMeLo-seq data; A.D., L.d.G., C.R.C., and M.V. generated the IF-FISH data; Y.W., T.Y.L., D.D.C., and P.M.L. generated the Strand-seq data; F.K.M. performed genome assembly and validation; F.K.M. and G.A.L. performed the centromere error correction; F.K.M. performed the centromere, phylogenetic, and statistical analyses with support from G.A.L. and E.E.E.; D.P. performed the Strand-seq data analysis; J.L., Y.S., and F.K.M. performed the SV and SNV analysis; J.W., A.N.R., and W.T.H. provided bioinformatics support for the centromere analysis; F.K.M. managed the data submission to dbGaP; and F.K.M., G.A.L., and E.E.E. drafted the manuscript. All authors read and approved the manuscript.

## Declaration of interests

E.E.E. is a scientific advisory board (SAB) member of Variant Bio, Inc.

## Web resources

OMIM, https://www.omim.org/

## Supplemental information

Document S1. Supplemental notes and Figures S1–S13

Table S1. HOR composition of chromosome 21 centromeres

Table S2. CDR methylation level quantification and comparison

Table S3. Summary information of the families with Trisomy 21

Table S4. Chromosome 21 centromere sizes of all individuals from the general population and the affected families

Document S2. Article plus supplemental information
