# Supplementary material for: Complete chromosome 21 centromere sequencing of families with Down syndrome
Source: Am J Hum Genet. 2026 Jun 17;113(7):1381–98. doi: 10.1016/j.ajhg.2026.05.010 (PMC13288774; doi:10.1016/j.ajhg.2026.05.010)
Supplement: Supplementary file 1 [file mmc1.pdf]

The American Journal of Human Genetics, Volume *113*

Supplemental information

Complete chromosome 21 centromere sequencing of families with Down syndrome

F. Kumara Mastrorosa, Alessia Daponte, Luciana de Gennaro, Shu-Cheng Chuang, Jiadong Lin, Yang Sui, David Porubsky, Keisuke K. Oshima, Julie Wertz, Allison N. Rozanski, William T. Harvey, Yanni Wang, Tiffany Y. Leung, Daniel D. Chan, Weiya He, Jordan Knuth, Gage H. Garcia, Marcelo Ayllon, Katherine M. Munson, Kendra Hoekzema, Peter M. Lansdorp, Claudia Rita Catacchio, Mario Ventura, Stephanie L. Sherman, Emily G. Allen, Tracie C. Rosser, Glennis A. Logsdon, and Evan E. Eichler

SUPPLEMENTAL NOTES

Strand-seq data analysis. Using Strand-seq data for proband AG16777, we were able to independently investigate the level of mosaicism and the breakpoint of the somatic translocation (**Figure S2**). We found that 8/64 or 12.5% of single-cell Strand-seq libraries carried the translocation. However, given this is a lower bound estimate, we consider it to be in line with what was described by Coriell biobank. The same data also allowed us to determine that the translocation breakpoints fall into a ~57 kbp region (chr21:34,572,930-34,630,265) on chr21 and into a 30 kbp region (chr22:31,794,966-31,825,441) on chr22 (**Figure S2**). These locations reside within cytogenetic chromosome bands 21q22.12 and 22q12.2, respectively, and overlap with the protein-coding genes *DOP1B* (MIM: 604803) (chr21) and *PATZ1* (MIM: 605165) (chr22).

Lastly, via Strand-seq, we were also able to study the recombination breakpoints in proband AG16777 (**Figure S13**). We identified 22 paternal and 33 maternal recombination breakpoints on the autosomes (**Figure S13D**), in line with what was recently described in a multigeneration pedigree family (maternal # range: 28-51; paternal # range: 22-34)[1]. We also observed similar features, such as a higher recombination rate in the mother and several breakpoints close to the telomere on the paternal side. We could not phase and study the breakpoints on chr21 due to an insufficient amount of data to resolve the additional complexity of three haplotypes. However, since significant reduction in recombination has been associated with nondisjoined homologs in T21 and other trisomies[2–6], there is a high chance that the maternally inherited chr21s were transmitted without crossing-over events.

Methylated CpG quantification. For each CDR of proband AG16777, we calculated the percentage of methylated CpGs (defined as having >50% methylated CpGs at the same position; **Material and methods**) across the region comprising the CDR pockets (from the start of the first CDR window to the end of the last one). The large maternal H1 CDR methylation level (46%) is moderately higher than both the short maternal H2 (15%) (Cramer's V = 0.30) and the paternal H3 (12%) (Cramer's V = 0.34), while for the short maternal H2 and paternal H3 percentages the difference is negligible (Cramer's V = 0.04). When comparing the methylation levels intergenerationally, we found that, among maternal centromeres, only the large H1 in the proband showed a weak increase (Cramer's V = 0.16) compared to the maternal counterpart (46% in proband, 33% in mother) (**Table S2**). A weak difference was also detected for the paternal centromere (Cramer's V = 0.15), whose methylation instead decreased in the proband (12% in proband, 22% in father) (**Table S2**).

Similar to what was found in the index trio, in proband 7210079.00, we found a moderately higher methylation level on the longer maternal 7210079.00-H3 (43%) when compared to the shorter maternal 7210079.00-H2 (19%) (Cramer's V = 0.25) and to its equivalent in the mother 7210079.20-H2 (22%) (Cramer's V = 0.32) (**Table S2**).

DiMeLo-seq signal quantification. We quantified the CENP-A DiMeLo-seq signal by calculating the fold enrichment of CENP-A DiMeLo-seq signal over IgG signal within each CDR, binned in 5 kbp windows. The proband's H1 centromere has a 445-fold enrichment of CENP-A over IgG signal (window size: 280 kbp), H2 a 358-fold enrichment (window size: 120 kbp), and H3 a 453-fold enrichment (window size: 135 kbp).

For duo 7210079, all three proband haplotypes showed enrichment of CENP-A within their CDRs: the large maternally inherited proband centromere 7210079.00-H3 has a 1,044-fold enrichment of CENP-A DiMeLo-seq signal over IgG signal (window size: 135 kbp), the paternally inherited 7210079.00-H1 has a 963-fold enrichment (window size: 155 kbp), and the short maternal 7210079.00-H2 an enrichment of 471-fold (window size: 90 kbp). As done for the methylation analysis, to analyze the longer maternally transmitted haplotype, we used the maternal assembly as a reference, since the centromere is not completely assembled in the proband, and we excluded the first proband CDR window since it falls within a sequence error in the assembly. Maternal

(7210079.20) data also show that the fold enrichment of CENP-A DiMeLo-seq signal over IgG signal inside the two haplotype CDRs has high values for both (H1: 1,052, window size: 130 kbp; H2: 786, window size: 150 kbp).

Variant analysis. We tested whether the genomes of probands with T21 have a higher number of variants compared to controls and a higher mutational burden. To perform this analysis, we identified from the long-read sequence data and assembly the total number of structural variants (SVs) and single-nucleotide variants (SNVs) in the families with T21 (**Material and methods**). The probands with T21 do not show a higher number of variants compared to their parents or a large number of population samples that have been sequenced and assembled in a similar fashion (**Figure S9**). Secondly, we focused on identifying rare SVs and *de novo* SNVs in proband AG16777 (the only proband for which both parents are available) using a recently described pangenome approach[7]. The number of rare SVs (*n*=166) and *de novo* SNVs (*n*=90) are comparable to those identified in autism families by Sui et al.[7] (ranging from 155 to 249 SVs and from 61 to 153 SNVs for matched ancestry controls), with similar methods indicating a comparable mutational burden and no excess of *de novo* mutations in the probands.

Population centromere analysis. We were not able to characterize both chr21 centromeres in 35 females and 30 males from the HPRC year 2 release. In these samples, one or none of the chr21 centromeres was assembled, or the haplotypes could not be reliably assigned to chr21 or chr13, which typically has much larger centromeres. This is common when the genomic contigs containing the centromeric sequences are not sufficiently extended to encompass the pericentromeric regions of both p- and q-arms and highlights the difficulty of sequencing and assembling large centromeres. As an example, two female samples (HG00320 and HG04184) carried a small chr21 centromere (228 kbp and 263 kbp, respectively), but their putative homologs were either incomplete or contained errors, with ambiguous assignments to chr21 or chr13. Some of these contigs contained large α-satellite arrays, but despite further analysis, we were unable to conclusively determine whether these putative homologs represented chr21, chr13, or a mix of the two centromeres. Consequently, they were excluded. It is worth noting though that even with two cases of extreme size asymmetry in the female population cohort, we would still have a significant difference in the number of cases observed in the two groups (population samples = 85; Fisher’s exact test p-value = 0.028).

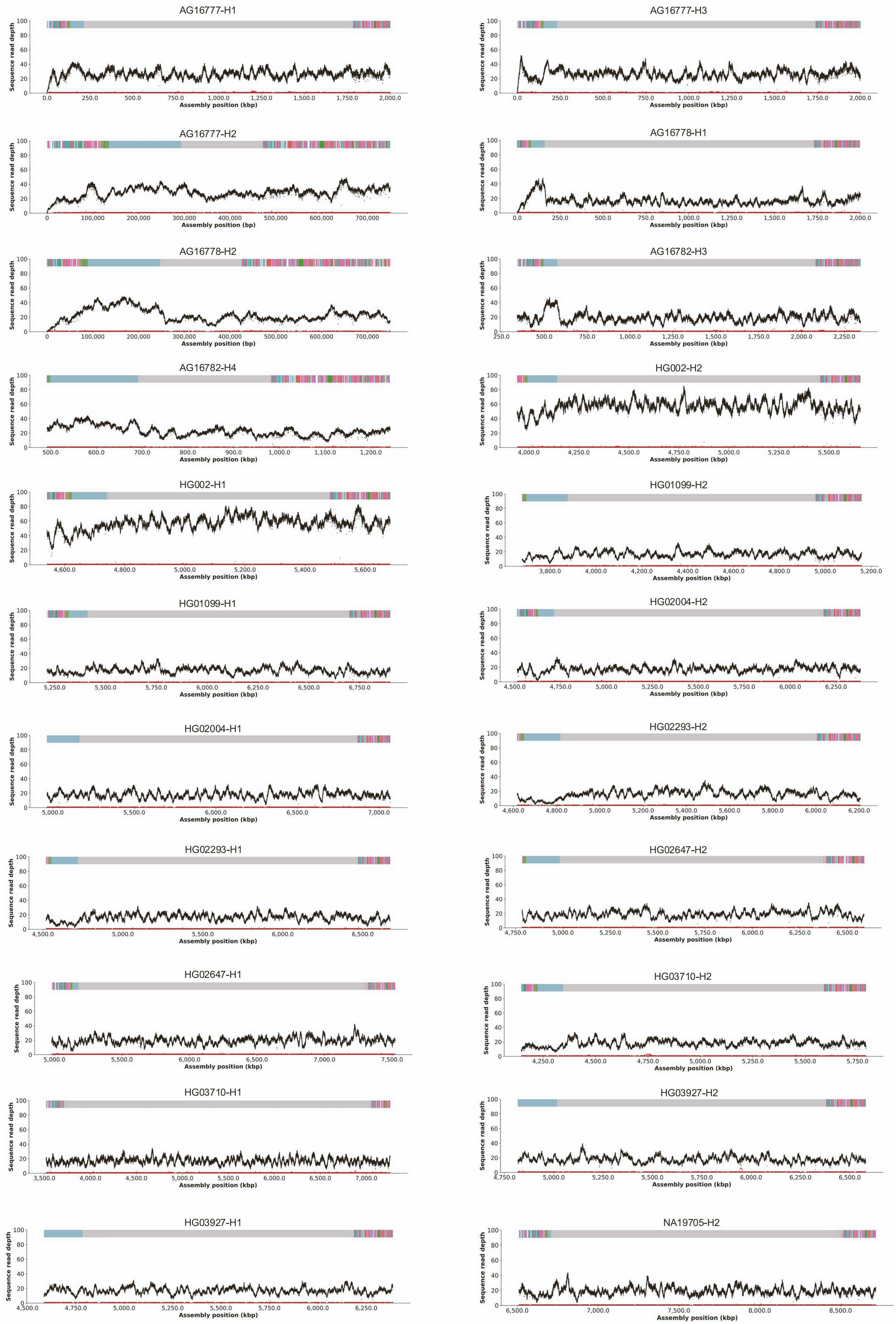

AG16777-H1
AG16777-H3
AG16777-H2
AG16778-H1
AG16778-H2
AG16782-H3
AG16782-H4
HG002-H2
HG002-H1
HG01099-H2
HG01099-H1
HG02004-H2
HG02004-H1
HG02293-H2
HG02293-H1
HG02647-H2
HG02647-H1
HG03710-H2
HG03710-H1
HG03927-H2
HG03927-H1
NA19705-H2
Sequence read depth
Assembly position (kbp)
Assembly position (bp)

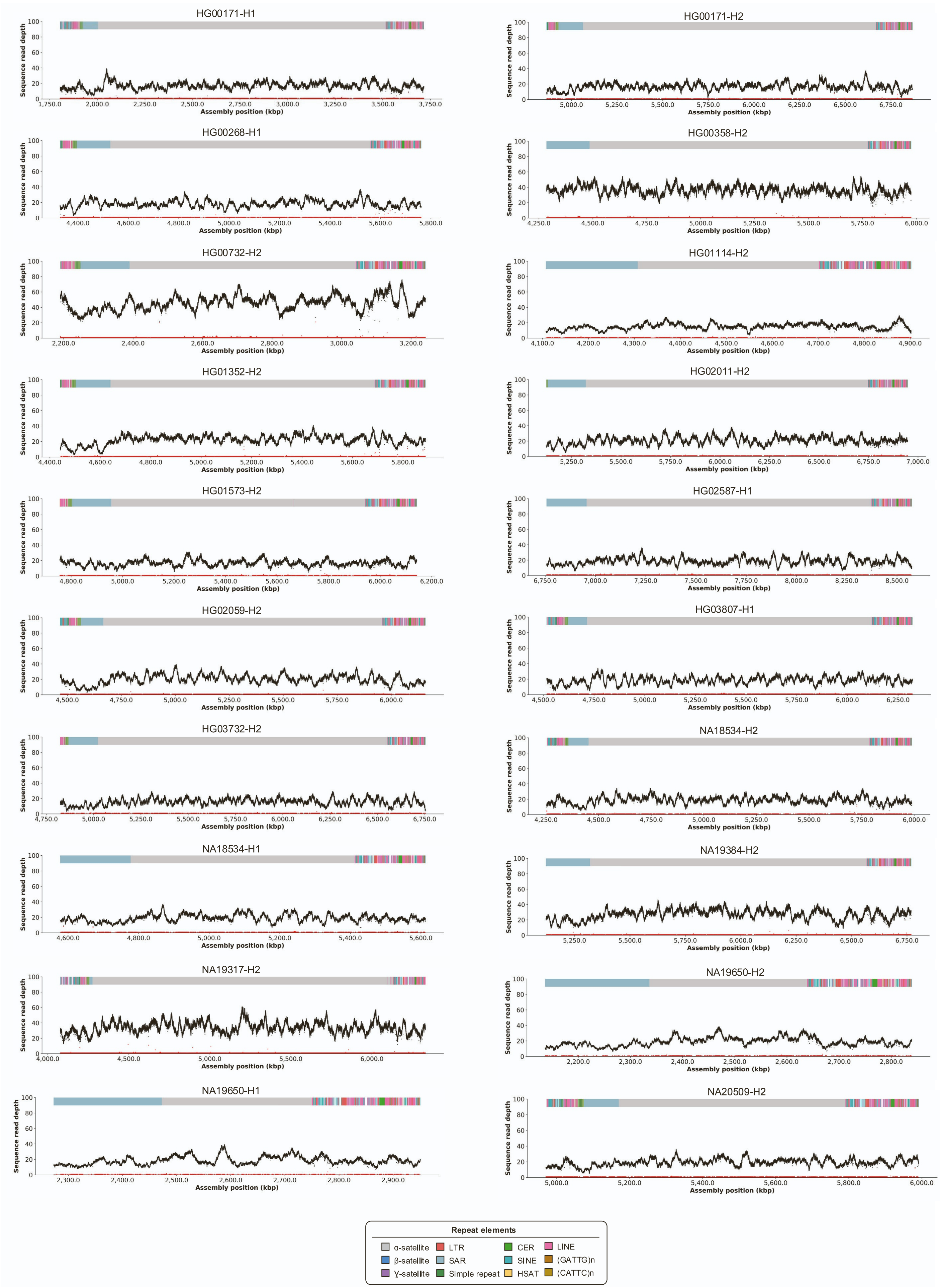

Figure S1. Centromere sequence validation. NucFreq validation of all chr21 centromeres described in family AG167 and population samples.

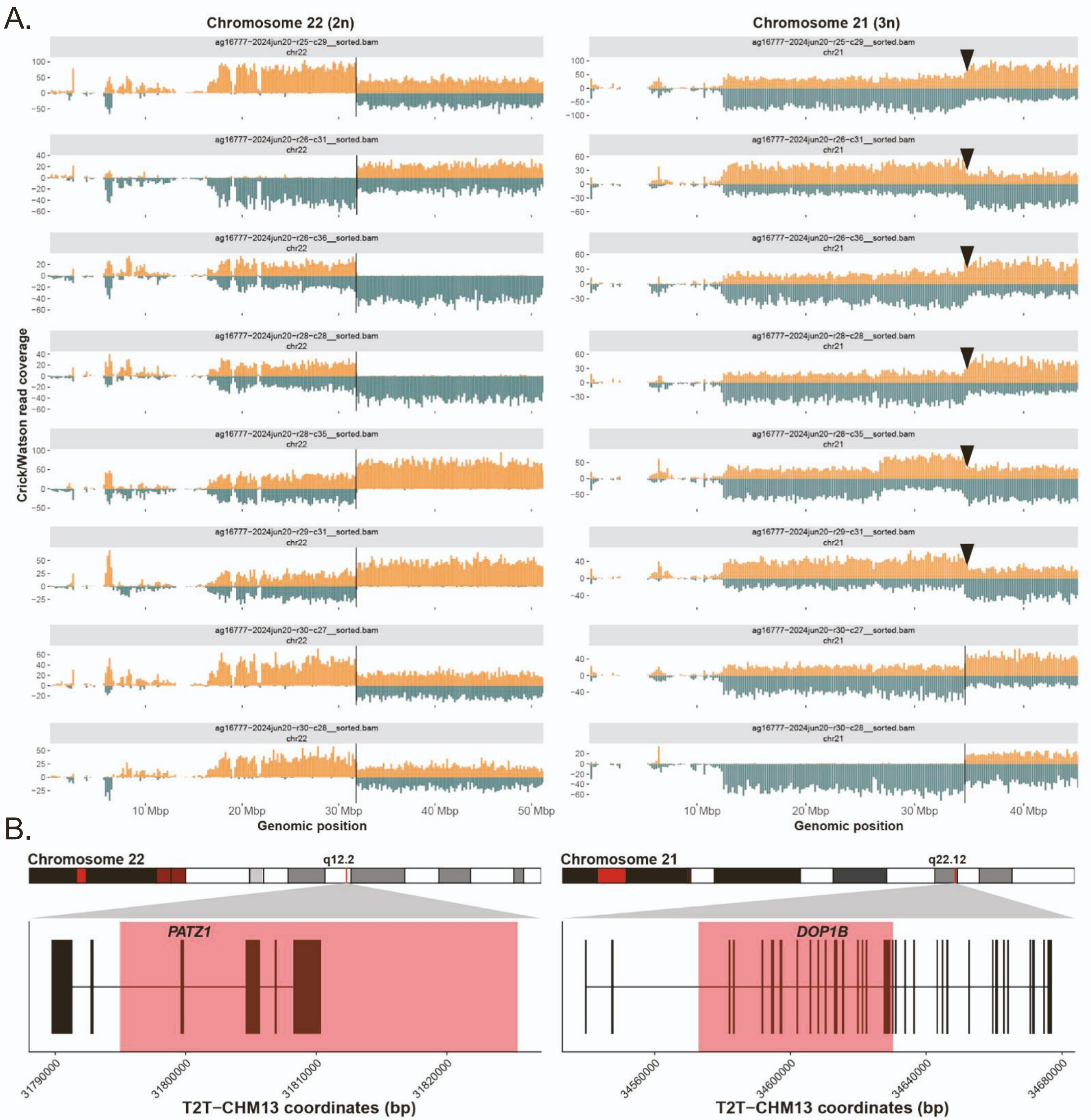

Figure S2. Strand-seq analysis of proband AG16777 identifies a mosaic translocation breakpoint between 21q22.12 and 22q12.2. A) Read-coverage profiles of Strand-seq data over both diploid (2n) chr22 (left) and triploid (3n) chr21 (right), summarized as binned (bin size: 200 kbp) read counts represented as colored bars above (orange; Watson read counts – W) and below (teal; Crick read counts – C) the midline. The translocation breakpoint is visible as strand state changes over the same location across all eight single cells. This is because, in these translocation informative cells, the translocated segment inherits the strand state from the original chromosomes it comes from. The defined strand state change is marked by vertical black lines as well as black arrowheads for triploid chr21 where these changes are difficult to detect due to the extra copy number. **B)** We predict the translocation breakpoint location at genomic position (with respect to T2T-CHM13 reference) chr21:34,572,930-34,630,265 (within the protein-coding gene *DOP1B*) and chr22: 31,794,966-31,825,441 (within the protein-coding gene *PATZ1*) for chr21 and 22, respectively. The predicted regions where translocation breakpoints likely occurred are highlighted by red rectangles, ~30 kbp in length for chr22 and ~57 kbp chr21.

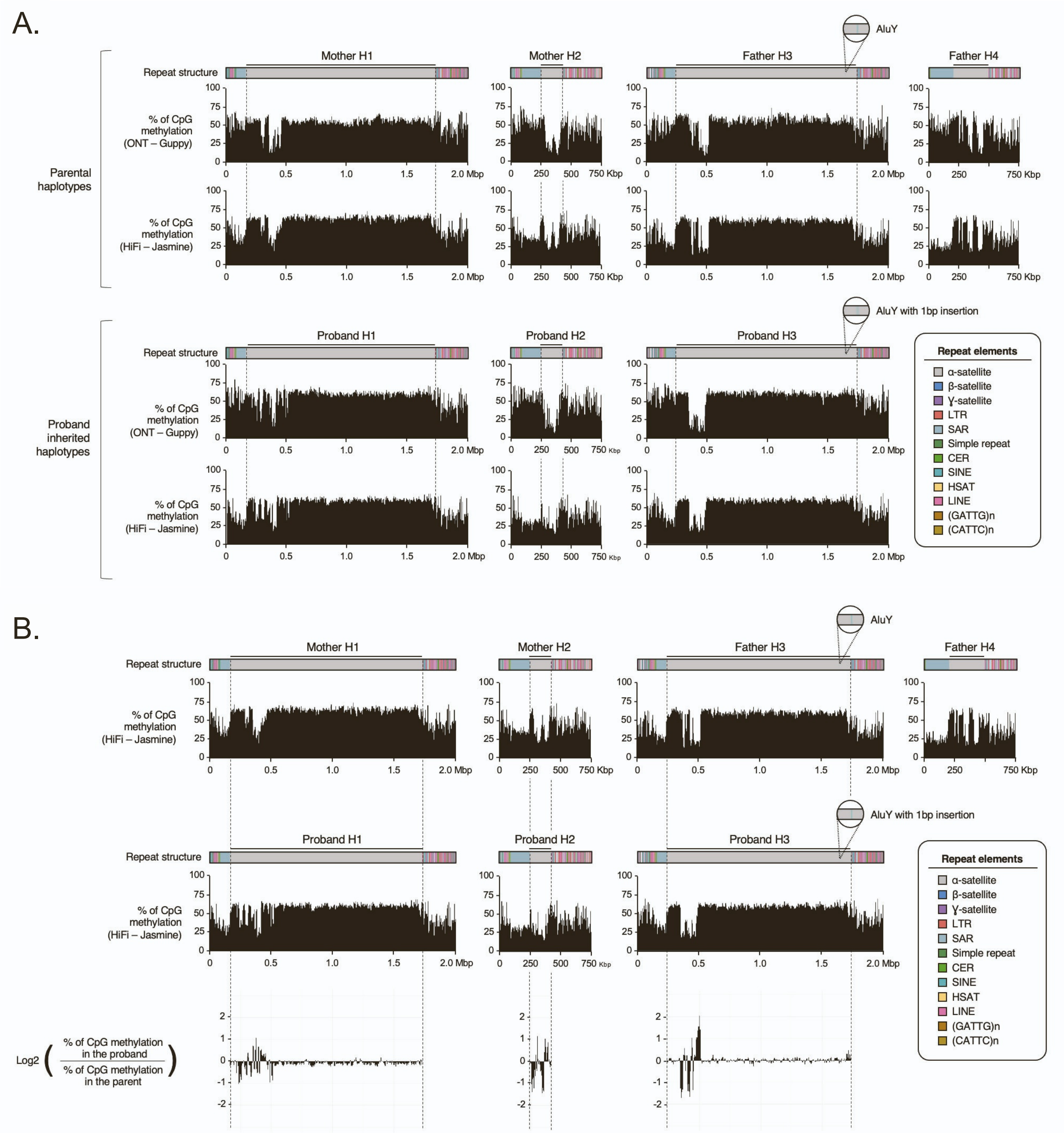

Figure S3. Centromeric methylation profiles comparison in trio AG167. A) Comparison of the ONT- and HiFi-based methylation profiles in all family AG167 chr21 centromeres. **B)** Comparison between proband and parental chr21 centromere methylation profiles based on the HiFi data.

AG167 Trio Immunoflourescence FISH

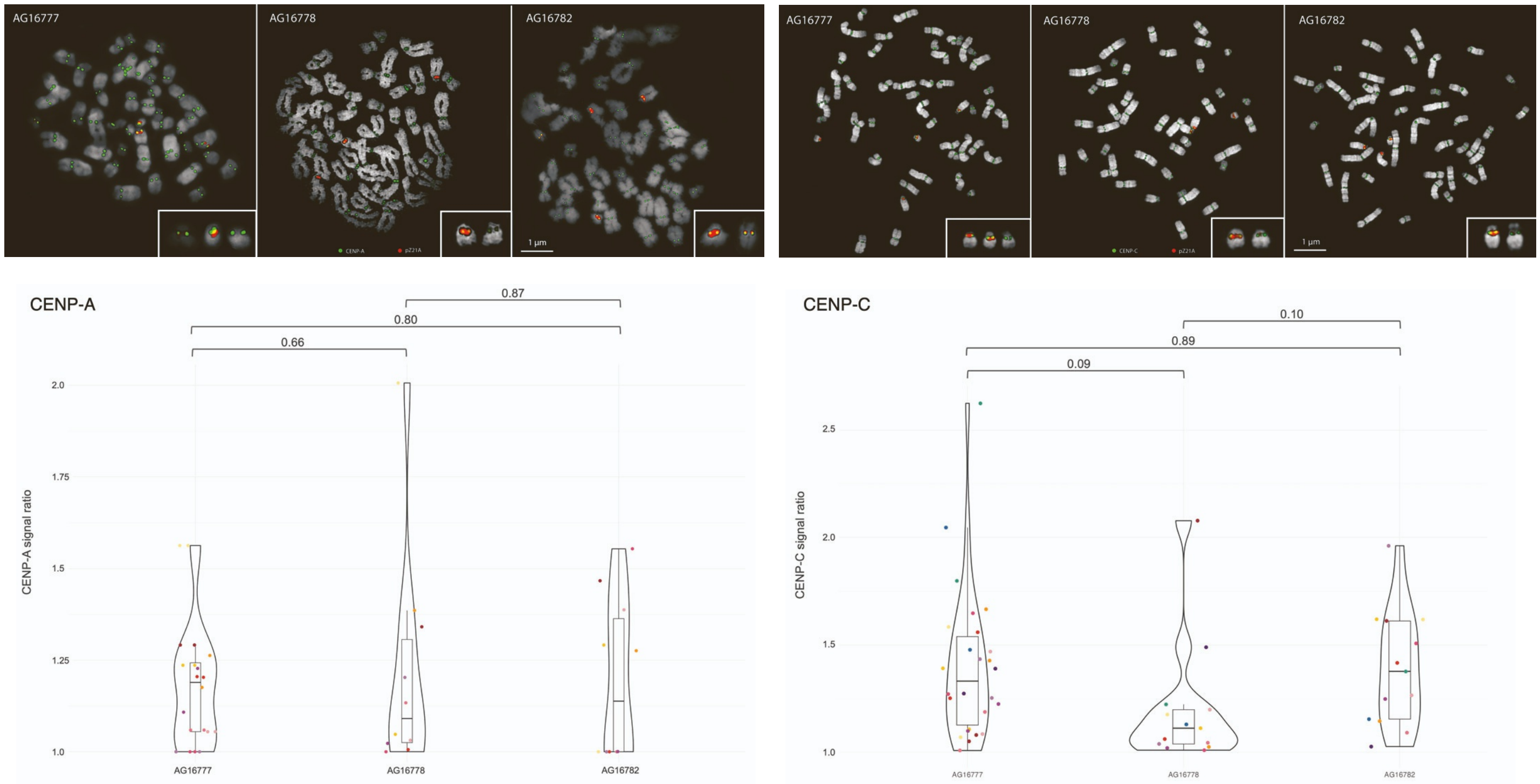

7210079 duo Immunoflourescence FISH

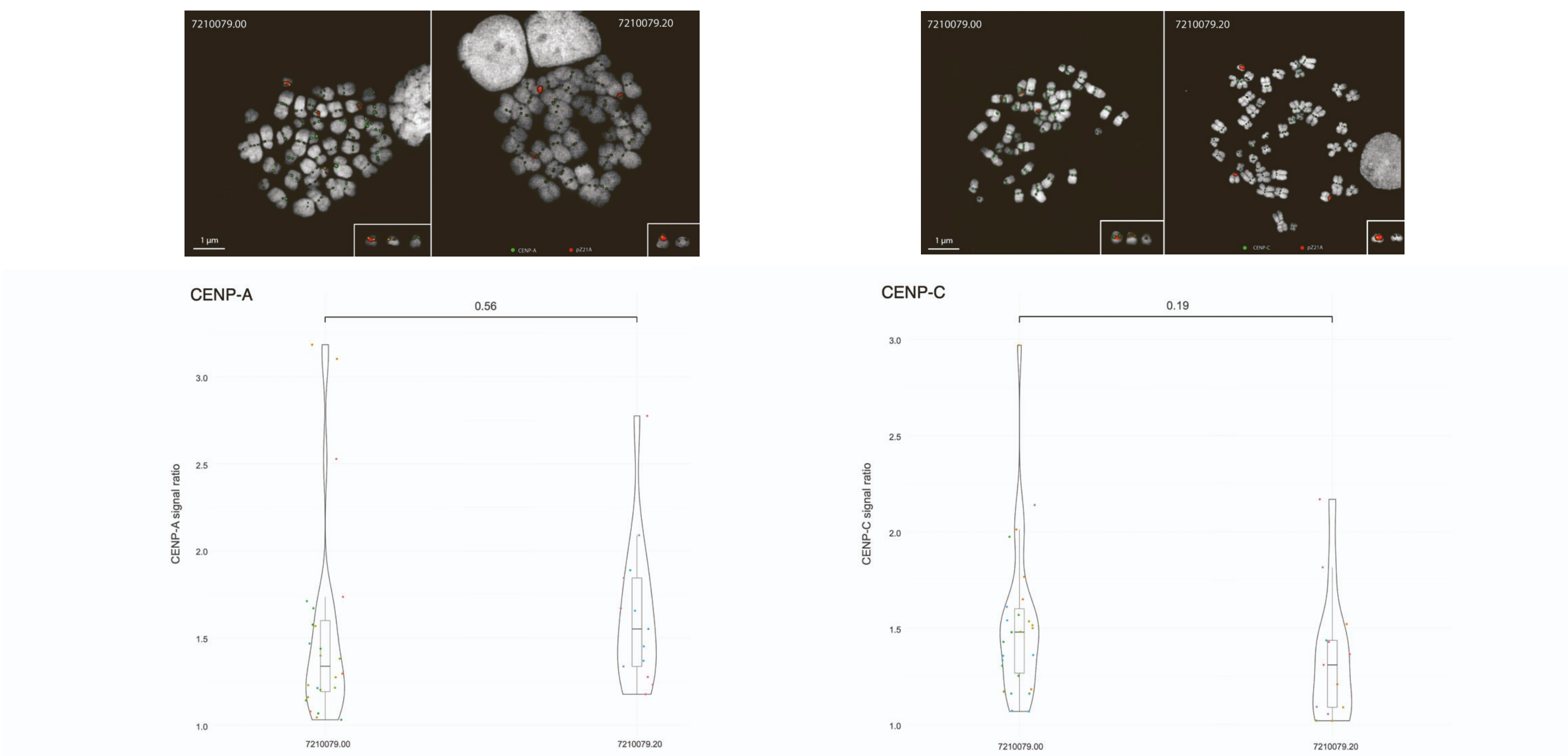

Figure S4. CENP-A and CENP-C detection and quantification in proband AG16777, mother AG16778, and father AG16782, proband 7210079.00, and mother 7210079.20. IF-FISH on metaphase chromosome spreads from all members of the AG167 family and duo 7210079, with fluorescent probes targeting the chr13/21 α-satellite array (red) and CENP-A/CENP-C (green). All chr21 are highlighted in the boxes. A weaker α-satellite array probe signal on one of the chr21 centromeres of proband and mother is due to the smaller centromeric α-satellite HOR array present in each of these individuals. The violin plots represent the CENP-A and CENP-C signal ratios for each member of the family. Each data point represents a metaphase from which the values of CENP-A/CENP-C protein signals were quantified. Each signal value was divided by the homolog lowest value to obtain the ratio and self-comparisons were removed. Signal ratio of 1 indicates an identical value between homolog signals. T-test p-values are indicated at the top for each comparison.

Additional complete centromeres in proband AG16777

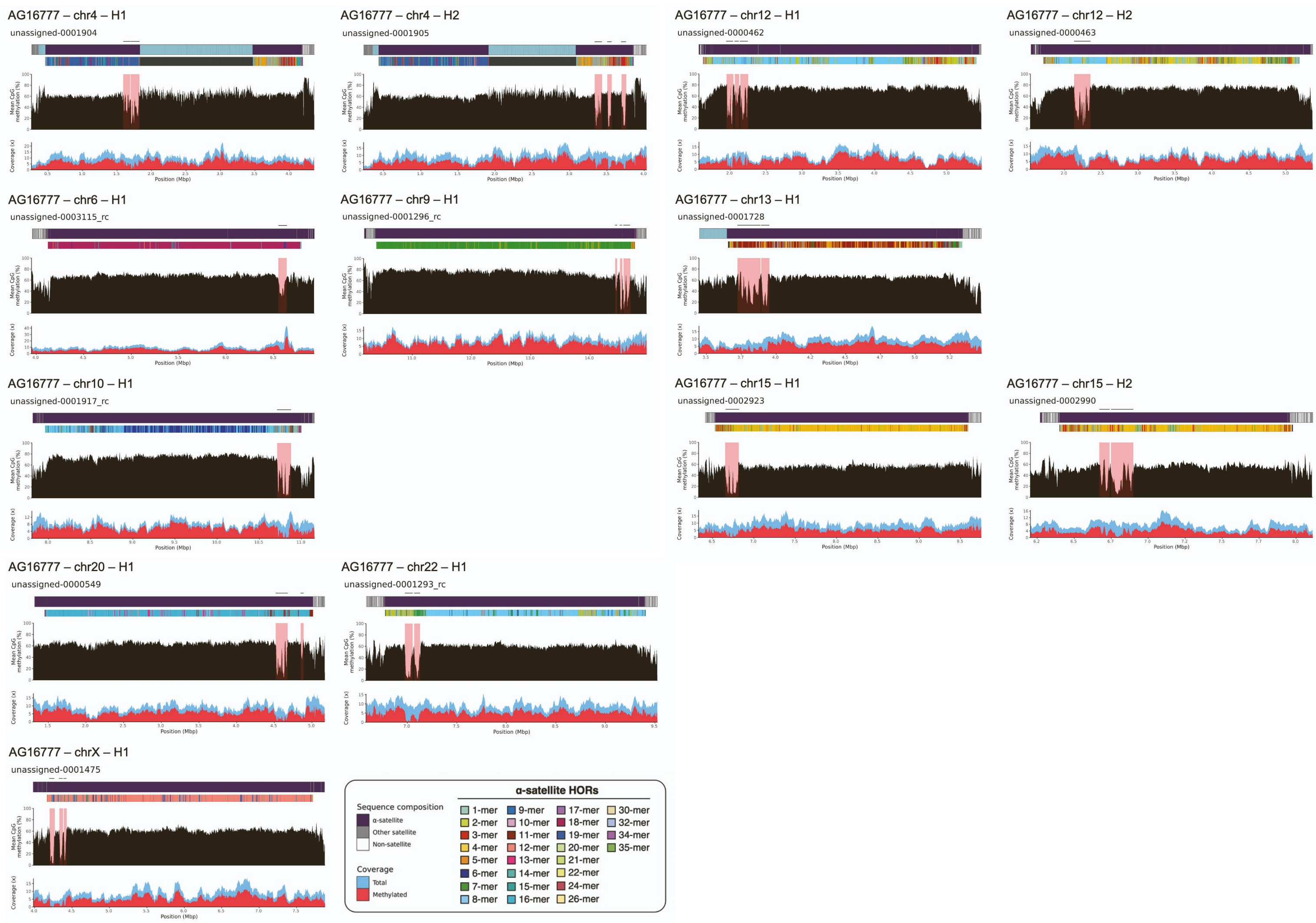

Additional complete centromeres in proband 7210079.00

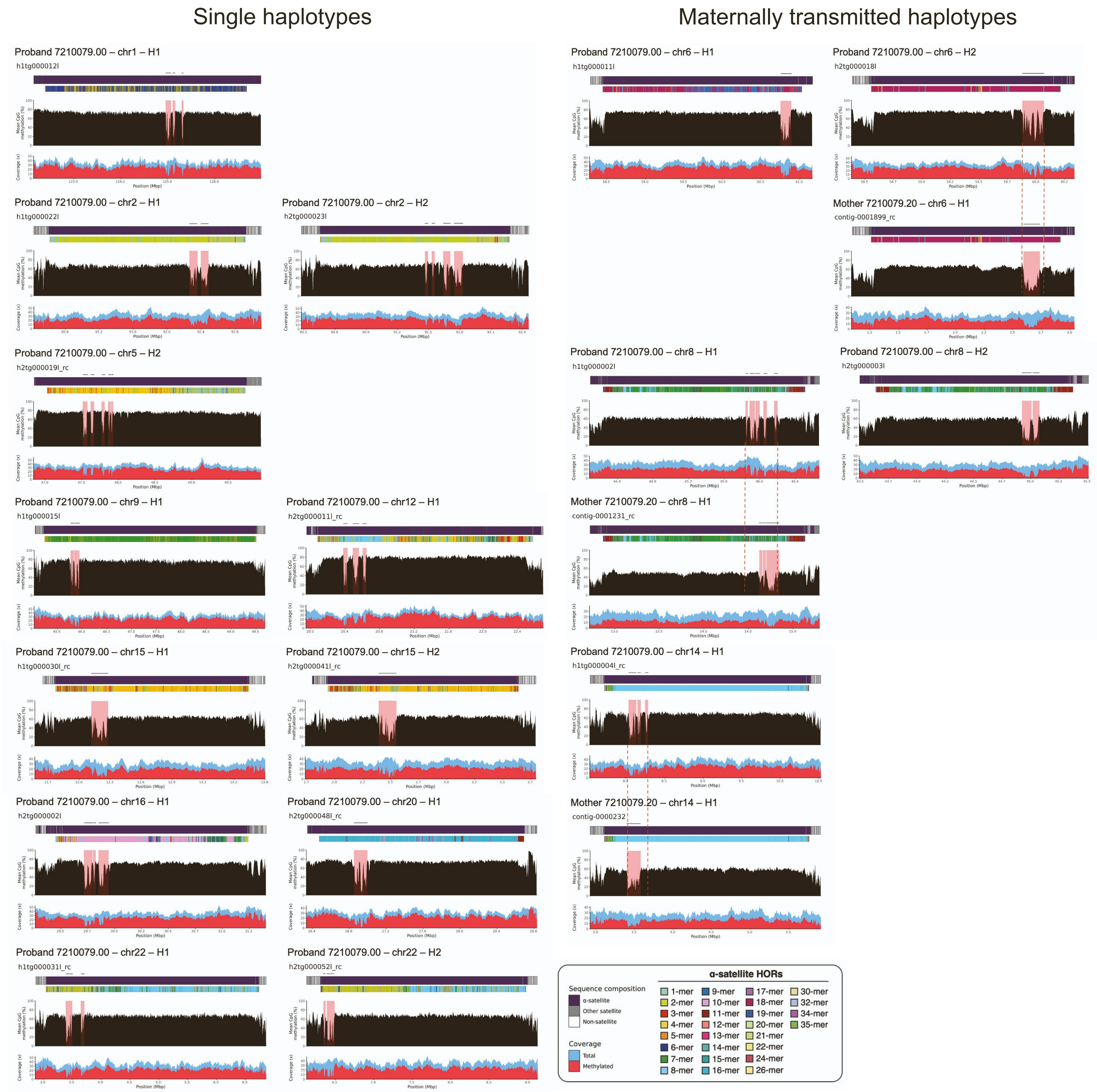

Additional complete centromeres in proband 7210079.20

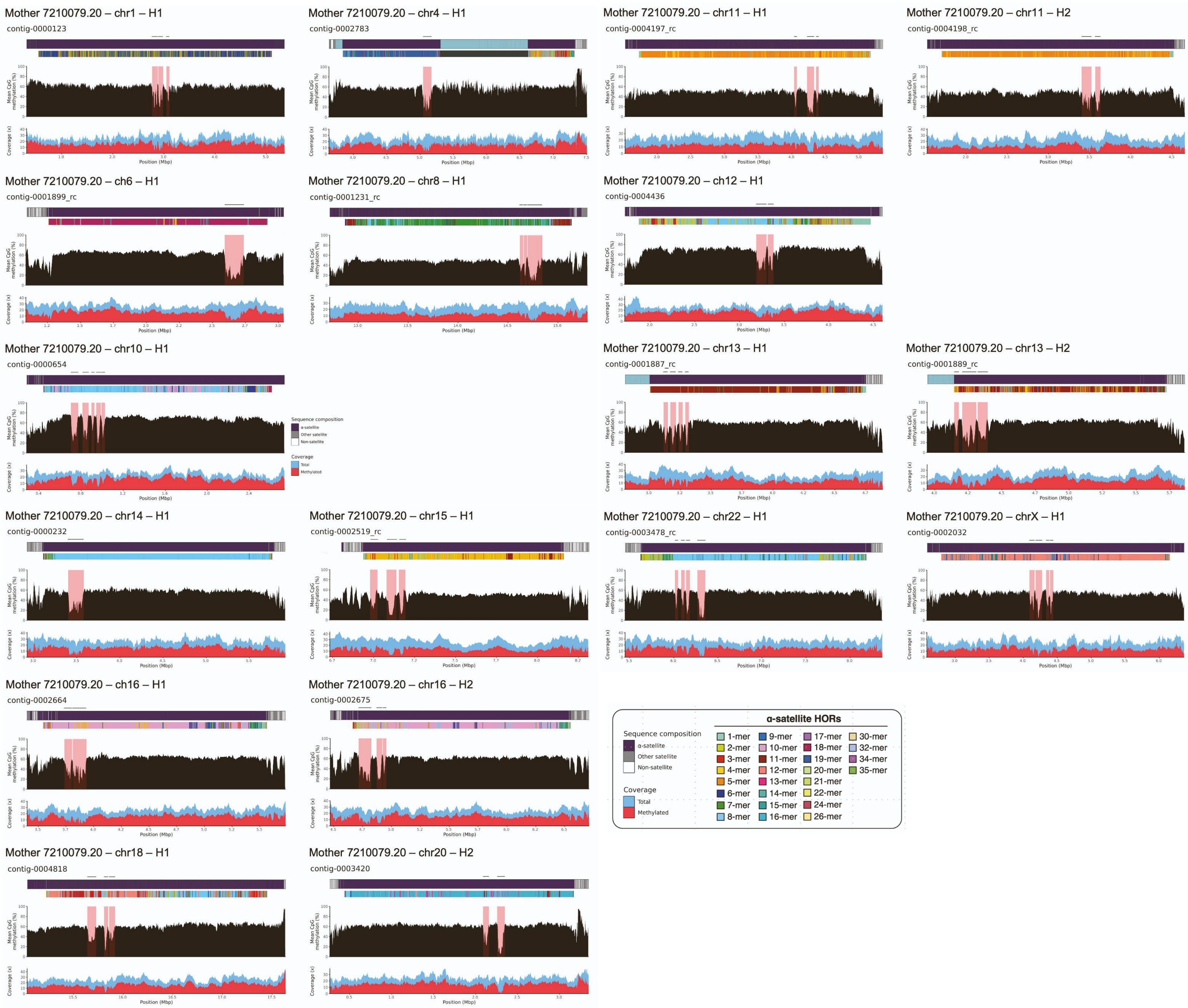

Figure S5. Characterization of non-chr21 centromeres. Additional complete centromeres in proband AG16777, proband 7210079.00, and mother 7210079.20.

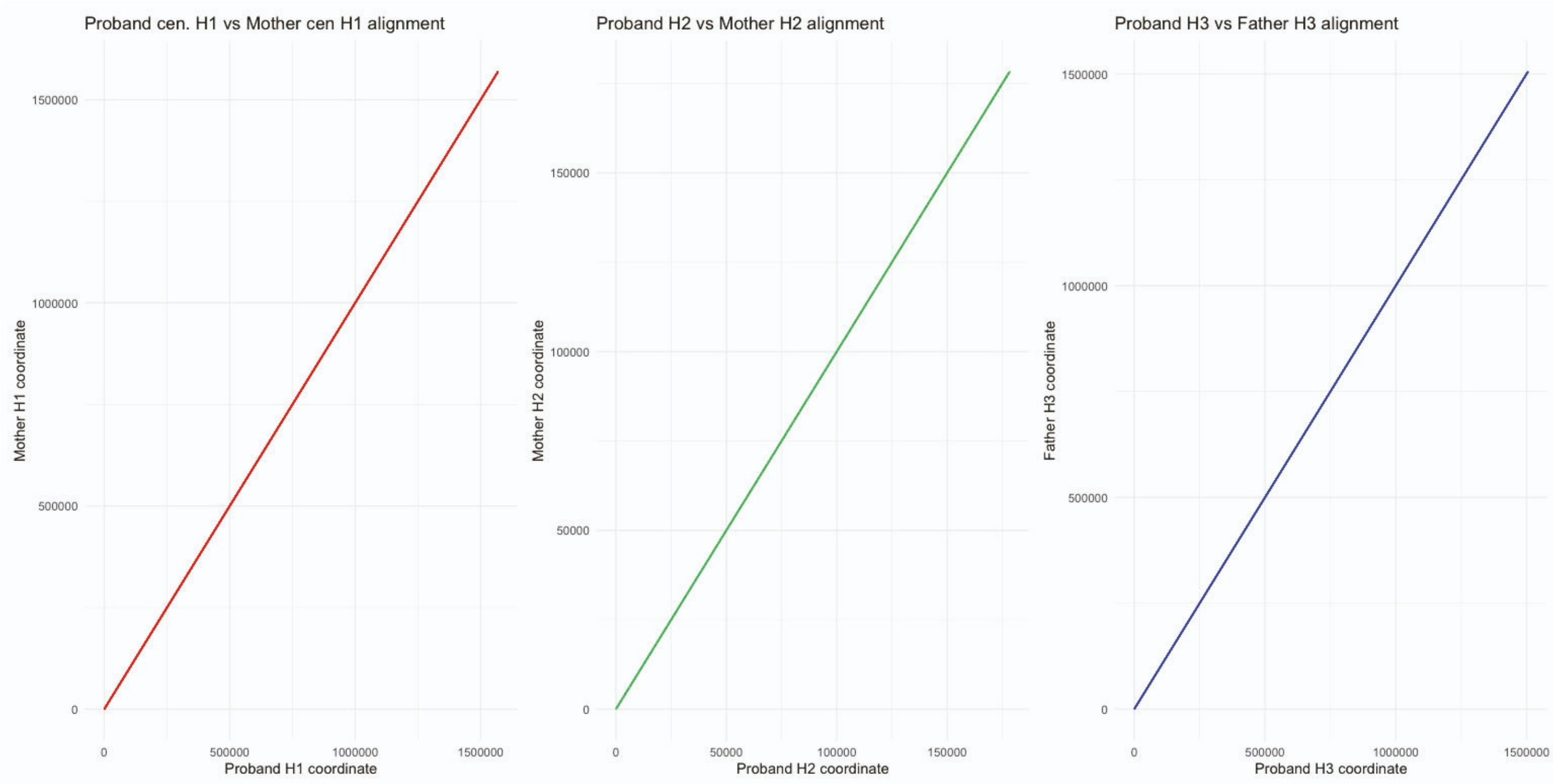

Figure S6. Parent-proband centromere sequences comparison. Dotplots comparing AG167 proband centromere sequences to their equivalents in parents. The plots represent the alignments of proband and parental centromeric α-satellite array sequences.

Query: Proband AluY insertion sequence + 10bp padding sequence on both sides
Subject: Father AluY insertion sequence + 10bp padding sequence on both sides

Score	Expect	Identities	Gaps	Strand
616 bits(333)	0.0	336/337(99%)	1/337(0%)	Plus/Plus

```
Query  1    AGTAGTTTCttttttttttttttttttttttttttttttGAGACGGAGTCTCGCTCTGTC  60
            ||||||||| |||||||||||||||||||||||||||||||||||||||||||||||||||
Sbjct  1    AGTAGTTTC-TTTTTTTTTTTTTTTTTTTTTTTTTTTTTGAGACGGAGTCTCGCTCTGTC  59

Query  61   GCCCAGGTCGGACTGCGGACTGCAGTGGCGCAATCTCGGCTCACTGCAAGCTCCGCTTCC  120
            ||||||||||||||||||||||||||||||||||||||||||||||||||||||||||||
Sbjct  60   GCCCAGGTCGGACTGCGGACTGCAGTGGCGCAATCTCGGCTCACTGCAAGCTCCGCTTCC  119

Query  121  CGGGTTCACGCCATTCTCCTGCCTCAGCCTCCCGAGTAGCTGGGACTACAGGCGCCCGCC  180
            ||||||||||||||||||||||||||||||||||||||||||||||||||||||||||||
Sbjct  120  CGGGTTCACGCCATTCTCCTGCCTCAGCCTCCCGAGTAGCTGGGACTACAGGCGCCCGCC  179

Query  181  ACCGCGCCCGGCTAATTTTTTGTATTTTTAGTAGAGACGGGGTTTCACCTTGTTAGCCAG  240
            ||||||||||||||||||||||||||||||||||||||||||||||||||||||||||||
Sbjct  180  ACCGCGCCCGGCTAATTTTTTGTATTTTTAGTAGAGACGGGGTTTCACCTTGTTAGCCAG  239

Query  241  GATGGTCTCGATCTCCTGACCTCATGATCCACCCGCCTCGGCCTCCCAAAGTGCTGGGAT  300
            ||||||||||||||||||||||||||||||||||||||||||||||||||||||||||||
Sbjct  240  GATGGTCTCGATCTCCTGACCTCATGATCCACCCGCCTCGGCCTCCCAAAGTGCTGGGAT  299

Query  301  TACAGGCGTGAGCCACCGCGCCCGGCCACCAAGTAGT  337
            |||||||||||||||||||||||||||||||||||||
Sbjct  300  TACAGGCGTGAGCCACCGCGCCCGGCCACCAAGTAGT  336
```

Figure S7. **Proband-father comparison of centromeric AluY sequences**. BLAST-based pairwise alignment of the AluY sequences inserted in AG167 proband H3 and father H3 centromere sequences.

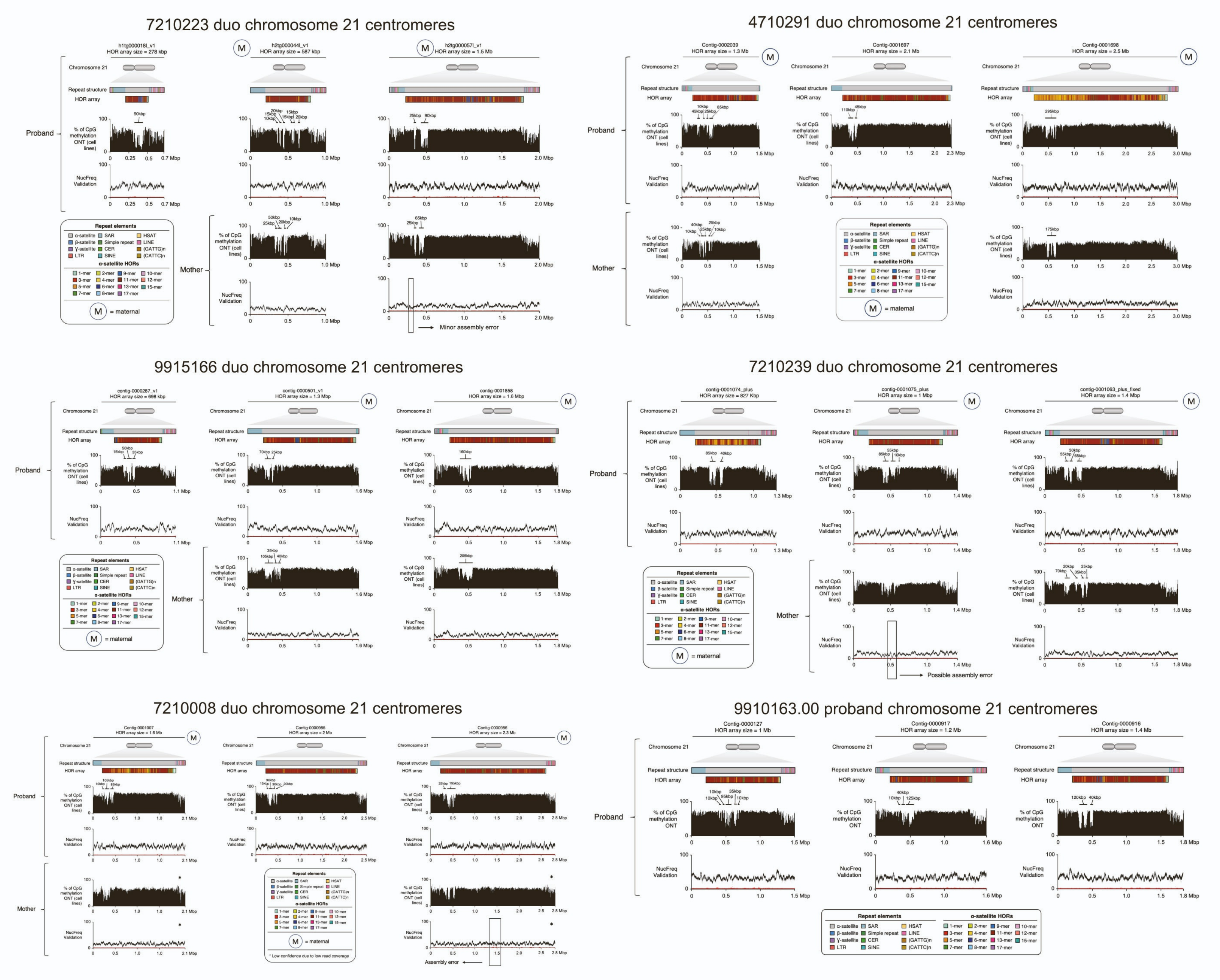

Figure S8. Centromere characterization in additional families with T21. Centromeres of individuals with T21 from families that do not exhibit extreme maternal centromere size asymmetry. Repeat structures, HORs, and methylation profiles are shown.

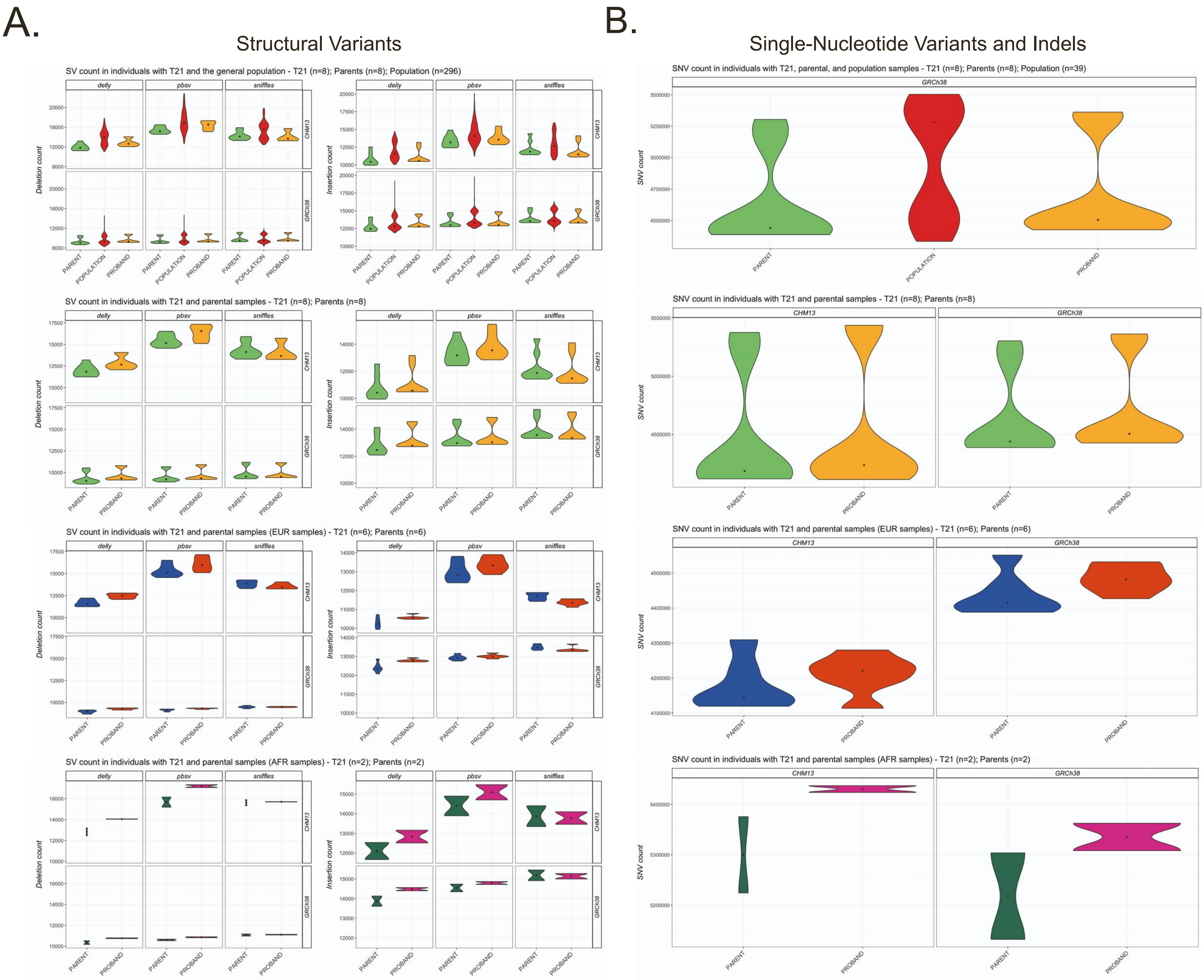

Figure S9. Variant comparison between probands and controls. A) Structural and **B)** single-nucleotide variants in probands, parents, and population samples.

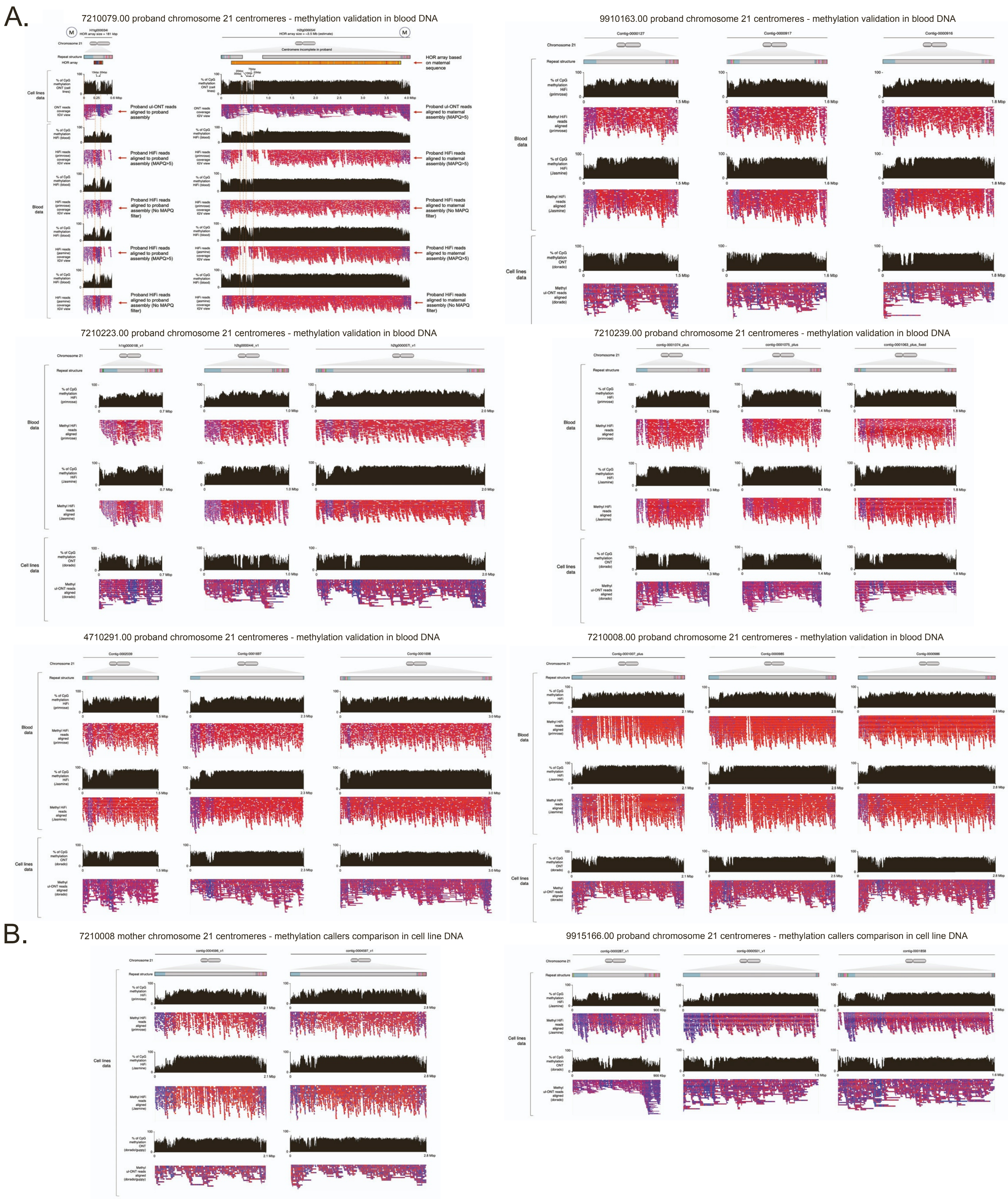

Figure S10. Comparison of chr21 centromere methylation profiles in blood and cell line data. A) Chr21 centromere methylation profiles detected from HiFi (blood DNA) and UL-ONT (lymphoblastoid cell line DNA) in probands. **B)** Methylation callers performance on cell line data.

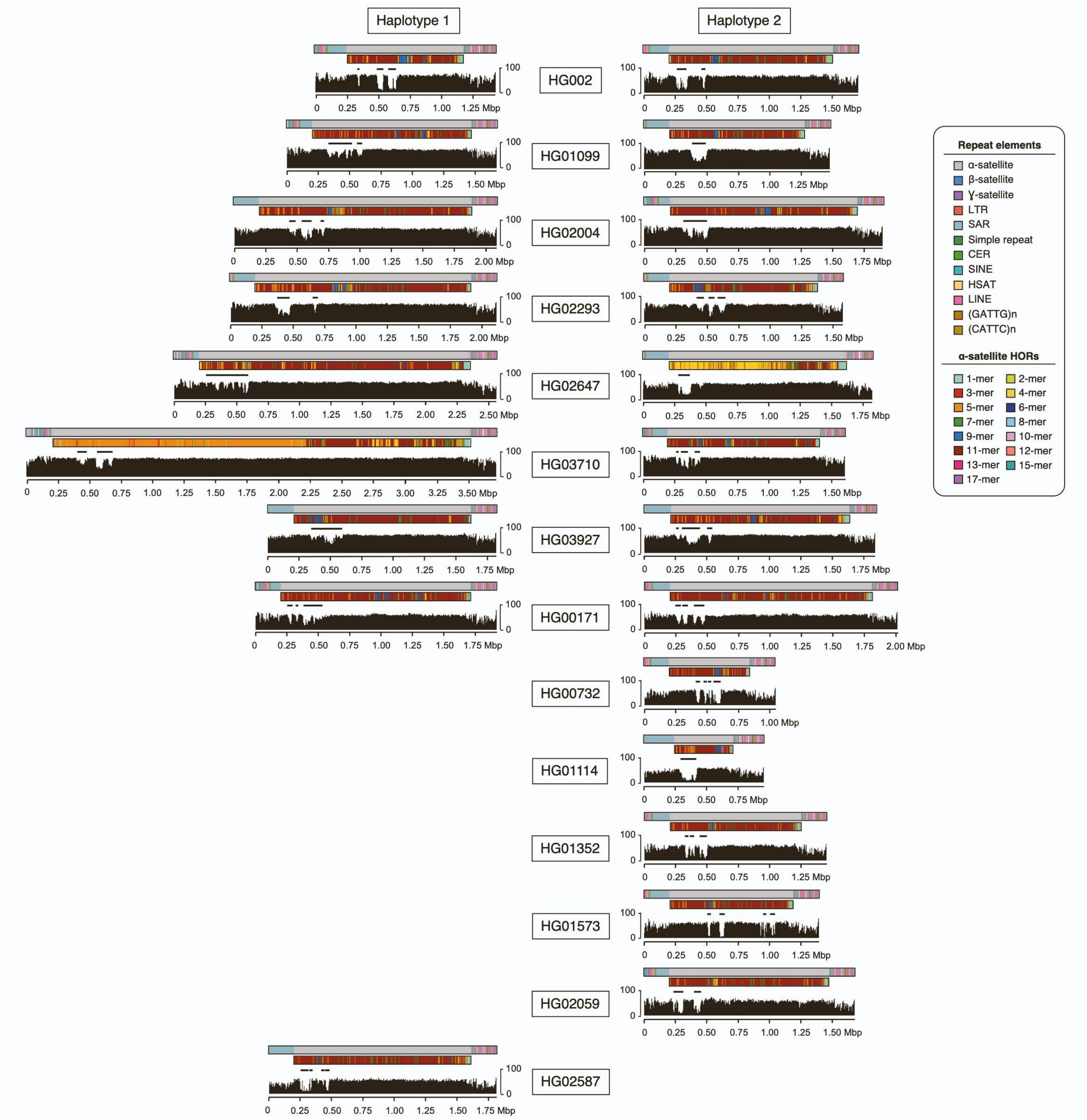

Figure S11. Extended version of Figure 4B. Methylation profiles and CDR positions in 22 population chr21 centromere haplotypes.

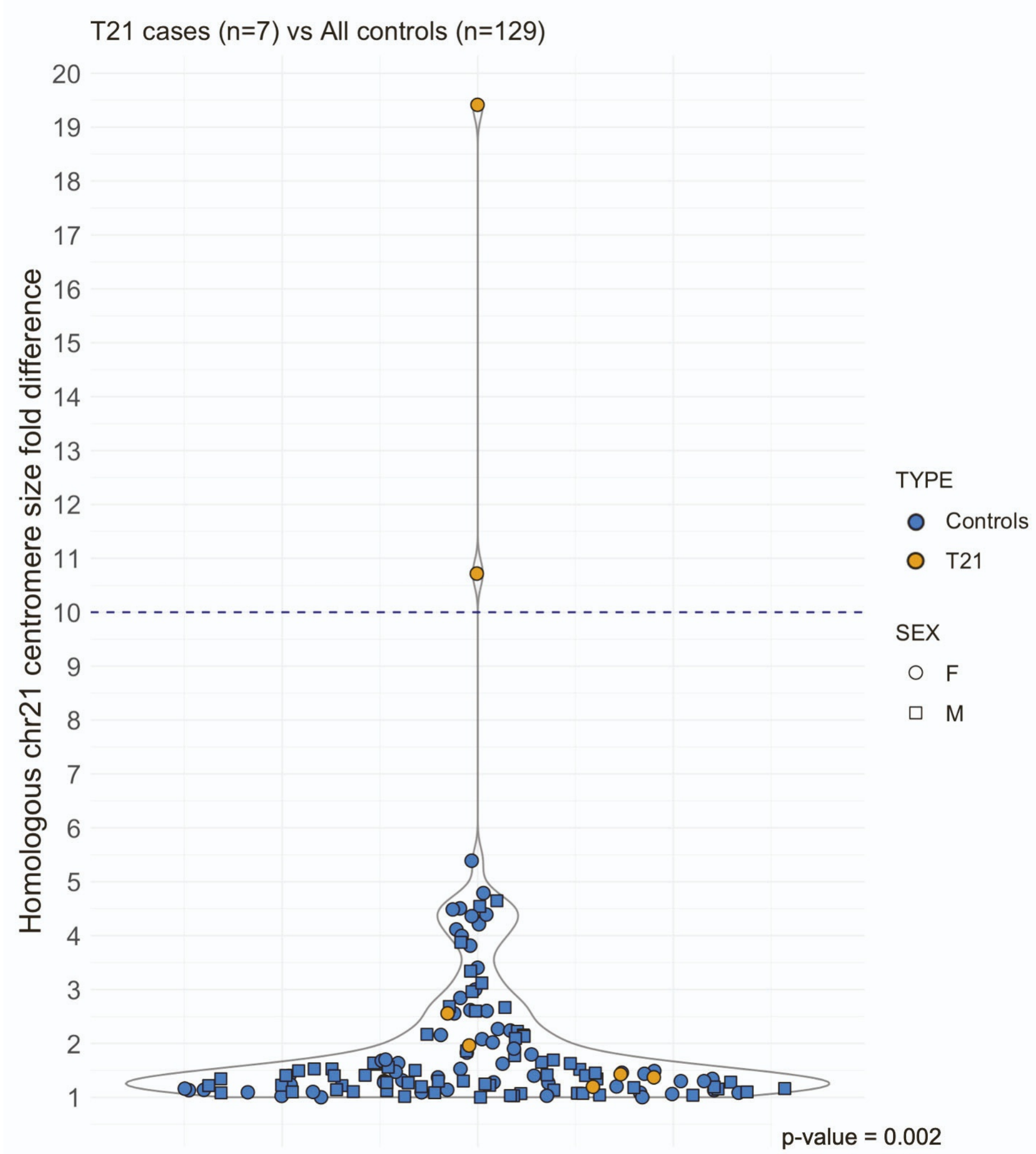

Figure S12. Homologous chr21 centromere size fold differences in T21 centromeres (only maternally inherited) and population samples. T21 (orange) and population (blue) samples are represented with different colors; sex is represented with different shapes. P-value indicates a significant difference in asymmetry cases between the two groups.

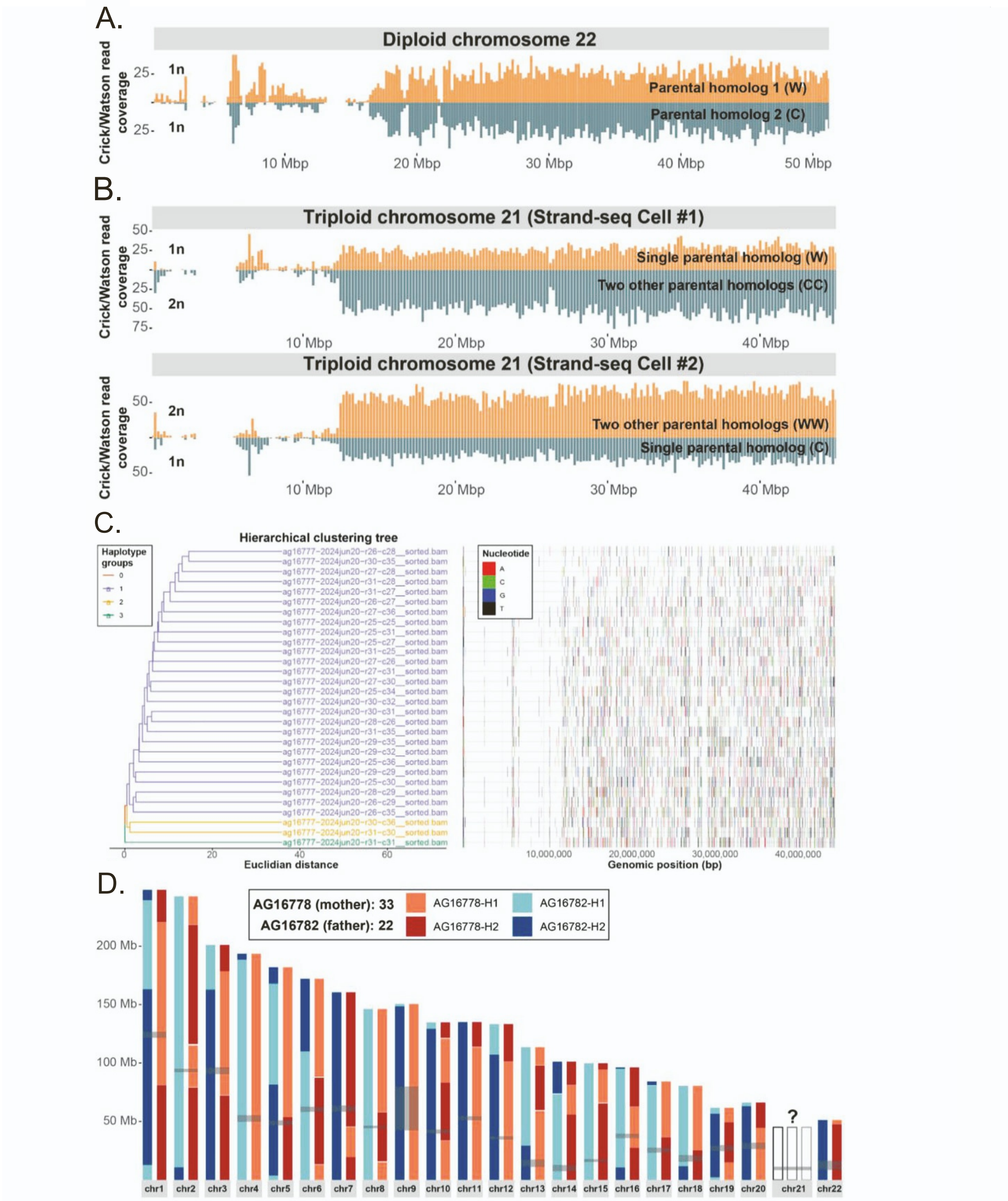

Figure S13. Strand-seq phasing of all chromosomes in proband AG16777, except for the triploid chr21.

A) Read-coverage profiles of strand-seq data over the diploid chr22, summarized as binned (bin size 200 kbp) read counts represented as colored bars above (orange; Watson read counts – W) and below (teal; Crick read counts – C) the midline. Here, equal coverage of Watson and Crick counts represents a single copy of inherited parental homologs for diploid (2n) chr22. **B)** Read-coverage profiles of strand-seq data over the triploid chr21 with the same binning used for panel A. Here, unequal coverage of Watson and Crick counts is represented by either a single copy (1n) of Watson or Crick reads accompanied by two copies (2n) Crick and Watson reads (WCC or WWC), respectively. **C)** Hierarchical clustering tree constructed based on concordance between 30 individual strand-seq haplotypes (left). Visualization of nucleotides present at each variable position among all 30 single-cell haplotypes (right). Given the sparsity of single-cell strand-seq data, there are a large number of gaps (white spaces) where there are no reads to capture the allele at a given variable position. **D)** A genome-wide overview of meiotic recombination breakpoints in proband (AG16777). Inherited segments of paternal homologs (H1, light blue; H2, dark blue) are on the left side of each chromosome while inherited segments of maternal homologs (H1, light red; H2, dark red) appear on the right side of each chromosome. Positions of detected recombination breakpoints are visible as changes from H1 to H2 segments and vice versa. Due to the sparsity of strand-seq data, we were not able to reconstruct chromosome-length haplotypes for trisomic chr21.

SUPPLEMENTAL REFERENCES

1. Porubsky, D., Dashnow, H., Sasani, T. A., Logsdon, G. A., Hallast, P., Noyes, M. D., Kronenberg, Z. N., Mokveld, T., Koundinya, N., Nolan, C., *et al.* Human de novo mutation rates from a four-generation pedigree reference. *Nature* 1–10 (2025)
2. Warren, A. C., Chakravarti, A., Wong, C., Slaugenhaupt, S. A., Halloran, S. L., Watkins, P. C., Metaxotou, C. & Antonarakis, S. E. Evidence for reduced recombination on the nondisjoined chromosomes 21 in Down syndrome. *Science* **237**, 652–654 (1987).
3. Sherman, S. L., Takaesu, N., Freeman, S. B., Grantham, M., Phillips, C., Blackston, R. D., Jacobs, P. A., Cockwell, A. E., Freeman, V., Uchida, I., *et al.* Trisomy 21: Association between reduced recombination and nondisjunction. *Am J Hum Genet* **49**, 608–620 (1991).
4. Hassold, T. & Sherman, S. Down syndrome: genetic recombination and the origin of the extra chromosome 21. *Clinical Genetics* **57**, 95–100 (2000).
5. Petersen, M. B. & Mikkelsen, M. Nondisjunction in trisomy 21: Origin and mechanisms. *Cytogenetics and Cell Genetics* **91**, 199–203 (2001).
6. Lamb, N. E., Yu, K., Shaffer, J., Feingold, E. & Sherman, S. L. Association between Maternal Age and Meiotic Recombination for Trisomy 21. *Am J Hum Genet* **76**, 91–99 (2005).
7. Sui, Y., Lin, J., Noyes, M. D., Kwon, Y., Wong, I., Koundinya, N., Harvey, W. T., Wu, M., Hoekzema, K., Munson, K. M., *et al.* Using the linear references from the pangenome to discover missing autism variants. *Nat Commun* (2026)
